# Rapid, ultra low coverage copy number profiling of cell-free DNA as a precision oncology screening strategy

**DOI:** 10.18632/oncotarget.21163

**Published:** 2017-09-22

**Authors:** Daniel H. Hovelson, Chia-Jen Liu, Yugang Wang, Qing Kang, James Henderson, Amy Gursky, Scott Brockman, Nithya Ramnath, John C. Krauss, Moshe Talpaz, Malathi Kandarpa, Rashmi Chugh, Missy Tuck, Kirk Herman, Catherine S. Grasso, Michael J. Quist, Felix Y. Feng, Christine Haakenson, John Langmore, Emmanuel Kamberov, Tim Tesmer, Hatim Husain, Robert J. Lonigro, Dan Robinson, David C. Smith, Ajjai S. Alva, Maha H. Hussain, Arul M. Chinnaiyan, Muneesh Tewari, Ryan E. Mills, Todd M. Morgan, Scott A. Tomlins

**Affiliations:** ^1^ Michigan Center for Translational Pathology, University of Michigan Medical School, Ann Arbor, MI, USA; ^2^ Department of Computational Medicine and Bioinformatics, University of Michigan Medical School, Ann Arbor, MI, USA; ^3^ Department of Pathology, University of Michigan Medical School, Ann Arbor, MI, USA; ^4^ Department of Urology, University of Michigan Medical School, Ann Arbor, MI, USA; ^5^ Department of Internal Medicine (Hematology/Oncology), University of Michigan Medical School, Ann Arbor, MI, USA; ^6^ Department of Biomedical Engineering, University of Michigan Medical School, Ann Arbor, MI, USA; ^7^ Department of Human Genetics, University of Michigan Medical School, Ann Arbor, MI, USA; ^8^ Department of Comprehensive Cancer Center, University of Michigan Medical School, Ann Arbor, MI, USA; ^9^ Department of Biointerfaces Institute, University of Michigan Medical School, Ann Arbor, MI, USA; ^10^ Division of Hematology-Oncology, University of California, Los Angeles and the Jonsson Comprehensive Cancer Center, Los Angeles, CA, USA; ^11^ The Parker Institute of Cancer Immunotherapy, San Francisco, CA, USA; ^12^ Departments of Radiation Oncology, Urology, and Medicine, University of California at San Francisco, San Francisco, CA, USA; ^13^ Takara Bio USA, Ann Arbor, MI, USA; ^14^ Medical Oncology, University of California, San Diego Moore's Cancer Center, San Diego, CA, USA; ^15^ Present address: Division of Hematology/Oncology, Feinberg School of Medicine, Northwestern University, Chicago, IL, USA

**Keywords:** cell-free DNA, precision oncology, prostate cancer, whole genome sequencing, copy-number analysis

## Abstract

Current cell-free DNA (cfDNA) next generation sequencing (NGS) precision oncology workflows are typically limited to targeted and/or disease-specific applications. In advanced cancer, disease burden and cfDNA tumor content are often elevated, yielding unique precision oncology opportunities. We sought to demonstrate the utility of a pan-cancer, rapid, inexpensive, whole genome NGS of cfDNA approach (PRINCe) as a precision oncology screening strategy via ultra-low coverage (~0.01x) tumor content determination through genome-wide copy number alteration (CNA) profiling. We applied PRINCe to a retrospective cohort of 124 cfDNA samples from 100 patients with advanced cancers, including 76 men with metastatic castration-resistant prostate cancer (mCRPC), enabling cfDNA tumor content approximation and actionable focal CNA detection, while facilitating concordance analyses between cfDNA and tissue-based NGS profiles and assessment of cfDNA alteration associations with mCRPC treatment outcomes. Therapeutically relevant focal CNAs were present in 42 (34%) cfDNA samples, including 36 of 93 (39%) mCRPC patient samples harboring AR amplification. PRINCe identified pre-treatment cfDNA CNA profiles facilitating disease monitoring. Combining PRINCe with routine targeted NGS of cfDNA enabled mutation and CNA assessment with coverages tuned to cfDNA tumor content. In mCRPC, genome-wide PRINCe cfDNA and matched tissue CNA profiles showed high concordance (median Pearson correlation = 0.87), and PRINCe detectable *AR* amplifications predicted reduced time on therapy, independent of therapy type (Kaplan-Meier log-rank test, chi-square = 24.9, *p* < 0.0001). Our screening approach enables robust, broadly applicable cfDNA-based precision oncology for patients with advanced cancer through scalable identification of therapeutically relevant CNAs and pre-/post-treatment genomic profiles, enabling cfDNA- or tissue-based precision oncology workflow optimization.

## INTRODUCTION

Clinical and commercial next-generation sequencing (NGS) based precision oncology strategies have expanded rapidly [1, 2]. Both targeted [3–8] and more comprehensive [9, 10] NGS assessment of frozen and archived formalin-fixed paraffin-embedded (FFPE) tissue samples have proven effective in identifying certain categories of clinically informative somatic DNA-based alterations, but tissue and re-biopsy requirements serve as considerable hurdles for widespread clinical implementation for identifying and tracking clinically relevant genomic alterations.

Myriad noninvasive (‘liquid biopsy’) approaches for identifying and tracking clinically relevant genomic alterations from cell-free DNA (cfDNA) have emerged as viable and potentially more broadly applicable alternatives to tissue-based assays using technologies including quantitative PCR (qPCR), digital droplet PCR (ddPCR), targeted DNA sequencing, and whole exome (WES) or whole genome sequencing (WGS) [2, 10–31]. Identifying a tractable, scalable precision oncology workflow with utility across patients with various advanced cancers, however, is still a substantial challenge given the variability of tumor-derived circulating cfDNA content, relevant genomic alterations, and frequent need for ultra-deep (e.g. > 10,000x), high-sensitivity sequencing in order to ensure detection (or absence) of clinically relevant alterations in pan-cancer cohorts [25, 32].

Genome-wide copy number profiles derived from low-pass cfDNA whole genome sequencing (WGS) are routinely used to detect large-scale aneuploidy events in clinical applications such as screening for fetal anomalies during pregnancy [33–36]. Multiple experiments have leveraged similar principles using low-pass cfDNA WGS to infer somatic whole-genome copy-number profiles in patients with advanced cancer, occasionally deploying higher depth disease-specific strategies for approximating cfDNA tumor content [22, 37–41]. However, these approaches often rely on disease specificity trade-offs that limit widespread prospective implementation [39]. Applicability across cancers, routine identification of actionable CNAs, correlation with comprehensive tissue based NGS profiling, and use as a precision oncology screen strategy have not yet been comprehensively addressed [40, 41]. Initiatives comparing comprehensive tissue-based molecular profiles to those obtained from cfDNA have also thus far been limited in size, particularly in metastatic castration resistant prostate cancer (mCRPC) [30, 31, 40].

Here, as part of an effort to facilitate precision medicine for all patients with advanced cancer, we propose a comprehensive approach deploying rapid, inexpensive, ultra-low pass cfDNA WGS as a broadly applicable potential screening strategy through: 1) directly identifying actionable CNAs, 2) informing needed sequencing depth for additional comprehensive/targeted cfDNA assessment (through cfDNA tumor content approximation) and 3) reserving ultra-deep cfDNA sequencing or tissue-based profiling for patients with low cfDNA tumor content. We show that with effective whole-genome coverage as low as 0.01× (< 100,000 single end reads) per sample on a benchtop Ion Torrent sequencer from as little as 10 pg of double-stranded DNA, we can recapitulate known whole-genome copy number profiles in cell lines and advanced prostate, colon, lung, and breast cancer patient samples, while retaining the ability to identify both focal and broad CNAs with megabase-level resolution. To confirm the utility of this screening approach to guide additional precision oncology assessment, we also paired this ultra-low-pass WGS with targeted multiplexed PCR based NGS of the same cfDNA, validating CNAs and identifying clinically relevant somatic mutation profiles at depth tuned by WGS-informed cfDNA tumor content approximation. Further, we directly compare cfDNA copy-number and mutational profiles with molecular profiles from synchronous or asynchronous tissue samples, highlighting high overall concordance and unique considerations for comprehensive precision oncology workflows, while exploring associations between putative cfDNA biomarkers and therapeutic outcomes in patients with mCRPC.

## RESULTS

### Rationale for a pan-cancer, rapid, inexpensive, ultra-low pass NGS cfDNA (PRINCe) approach to guide precision oncology

**Figure 1:**
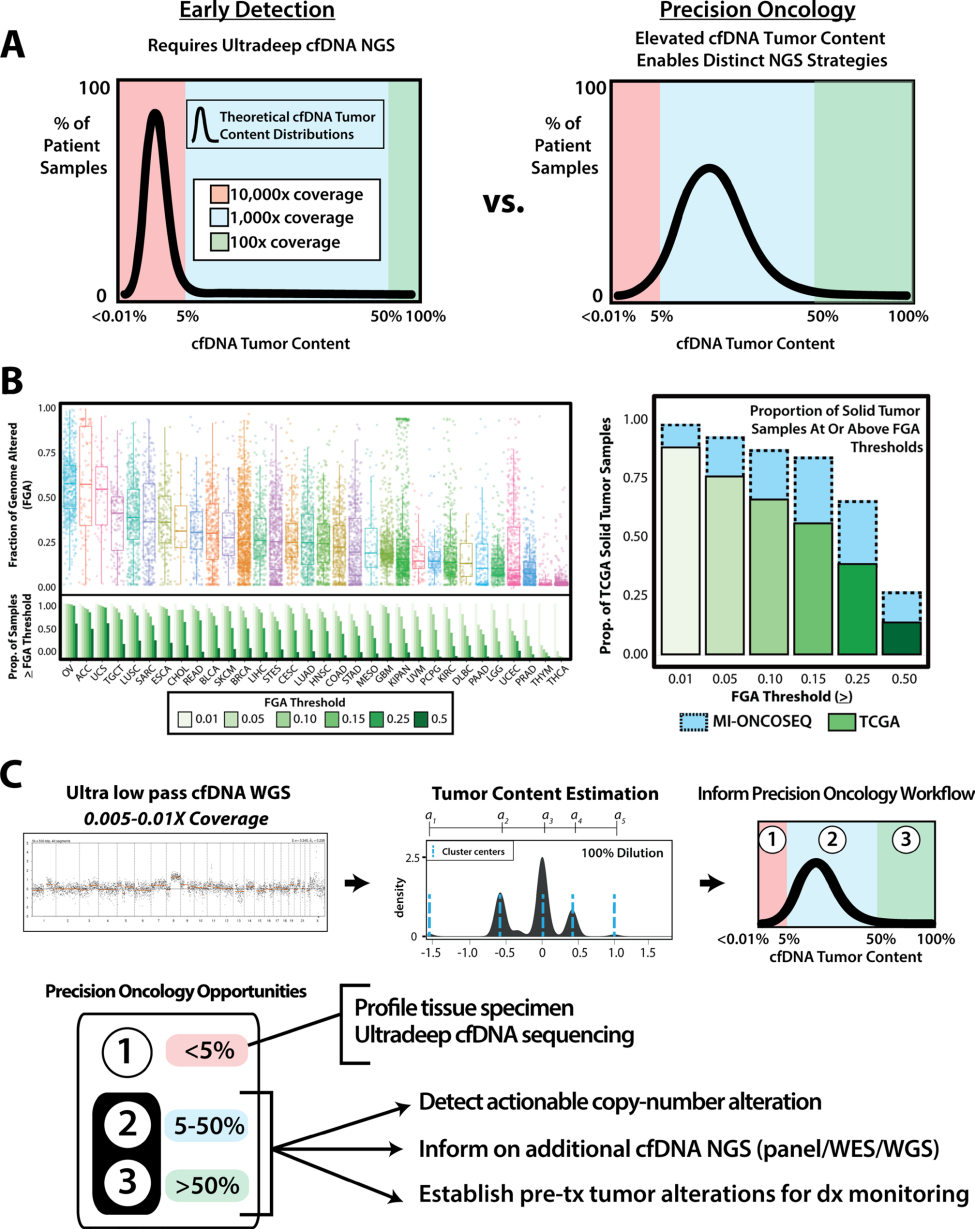
Leveraging tumor-derived cfDNA distribution in advanced cancer to develop a pan-cancer, rapid, inexpensive, ultra-low pass whole genome next generation sequencing (NGS) cfDNA precision oncology workflow (PRINCe) (**A**) Theoretical cfDNA tumor content distributions and typical next-generation sequencing (NGS) coverage requirements for mutation profiling are presented for early detection (left) and precision oncology in advanced disease (right) applications. In early detection context, the majority of cfDNA samples are expected to have a low proportion of tumor-derived cfDNA fragments (e.g., < 5%), whereas advanced cancers have an elevated proportion of tumor-derived cfDNA. Tumor content requiring ultra-deep, extreme-fidelity (e.g. 10,000x coverage) targeted sequencing are shaded red, while those amenable to targeted sequencing on larger panels or whole-exome/whole-genome (WES/WGS) are shaded blue and green, respectively. (**B**) Copy number alterations (CNAs) are frequent across human cancers. The fraction of the genome altered (FGA, see Methods) by CNAs in 11,576 The Cancer Genome Atlas (TCGA) samples from 32 solid tumor types is shown across multiple thresholds (overall cohort on left, individual tumor types on right). Increased FGA in a cohort of 129 advanced/metastatic cancers (prostate, kidney, lung and breast cancers) subjected to exome sequencing in the MI-ONCOSEQ program (plotted on the right panel) is seen in comparison to the TCGA cohort, consistent with increasing frequency of CNAs in advanced/metastatic cancers. (**C**) Schematic for pan-cancer, rapid, inexpensive, ultra-low pass NGS cfDNA workflow (PRINCe). Segmented copy-number calls from ultra-low-pass cfDNA whole-genome sequencing are generated, followed by CNA-clustering based tumor content approximation to inform on precision oncology management. In patients with sufficient tumor content by PRINCe (e.g. > 5–10%), CNA profiles may directly guide treatment (if focal targSupplementary Table alterations are identified), enable routine panel, WGS, or WES based cfDNA NGS tuned to tumor content, as well as establish pre-treatment (tx) CNA profiles for disease (dx) monitoring post-therapy. More costly ultra-deep, extreme fidelity cfDNA and tissue based profiling can thus be reserved for patients with low cfDNA tumor content.

The major impetus for ultra-deep, high sensitivity cfDNA profiling in precision oncology is the need for robust sensitivity and specificity for somatic alterations detection at extremely low cfDNA tumor content [42]. While many cfDNA-based detection approaches thus rely heavily on targeted, ultra-sensitive methodologies, many patients with elevated tumor burden or metastatic treatment refractory cancer—where precision oncology NGS is most commonly employed—have relatively high cfDNA tumor contents of 5–50% [22, 25, 42] (Figure 1A). If tumor-derived cfDNA characteristics could be rapidly leveraged to approximate tumor content and potentially identify clinically relevant alterations across cancer types, unique and potentially more optimized precision medicine strategies may be achievable. Given that somatic copy-number alterations (CNAs) are pervasive in cancer [43] and somatic copy-number burden may be an important marker for aggressive or treatment-resistant disease [44], we first assessed the prevalence of extended copy-number burden in a pan-cancer TCGA cohort using 11,576 copy number profiles from 32 tumor types (Figure 1B). Overall, 56% of tumors had elevated copy-number burden (defined by having > 15% fraction of the genome altered [FGA]), with FGA increasing with pathologic tumor stage, tumor grade and clinical stage (Supplementary Figure 1). Importantly, per-sample FGA was also increased in a cohort of advanced/metastatic tumors (*n* = 129) profiled as part of the MI-ONCOSEQ project [45] compared to the TCGA cohort, with 81% of Mi-ONCOSEQ profiled tumors having > 15% FGA (Figure 1B). As CNAs can be robustly detected at substantially lower sequencing coverage (and cost) than typically required for somatic mutation calling in genome-wide or targeted pan-cancer workflows, we sought to exploit genome-wide CNAs as a biomarker through a pan-cancer, rapid, inexpensive, ultra-low pass NGS cfDNA (PRINCe) precision oncology screening approach, which has the potential to directly inform precision oncology workflows through genome-wide CNA detection and tumor content approximation (Figure 1C).

### Validation of ThruPLEX cfDNA WGS for Ion torrent benchtop sequencers and cfDNA tumor content approximation

Validation of cfDNA WGS using a three hour ThruPLEX RGP-0003 WGA single tube library construction approach (compatible with ≤ 50pg double stranded DNA) for rapid sequencing on Ion Torrent benchtop sequencers was carried out on 10 normal control cfDNA samples, all of which displayed high sequencing coverage uniformity (> 90%) (Supplementary Table 1 and Supplementary Results). *In vitro* dilution experiments of sheared genomic DNA for VCaP (prostate cancer) and UMUC-5 (bladder cancer) cell lines confirmed our ability to leverage Ion Torrent cfDNA WGS for recapitulation of whole-genome copy number profiles and detection of therapeutically relevant focal amplifications (including *AR* and *EGFR* amplifications), with high observed concordance with orthogonal targeted and genome-wide copy-number profiles at tumor contents as low as 5% (see Supplementary Figures 2 and 3, Methods, Supplementary Results) [46, 47].

**Figure 2:**
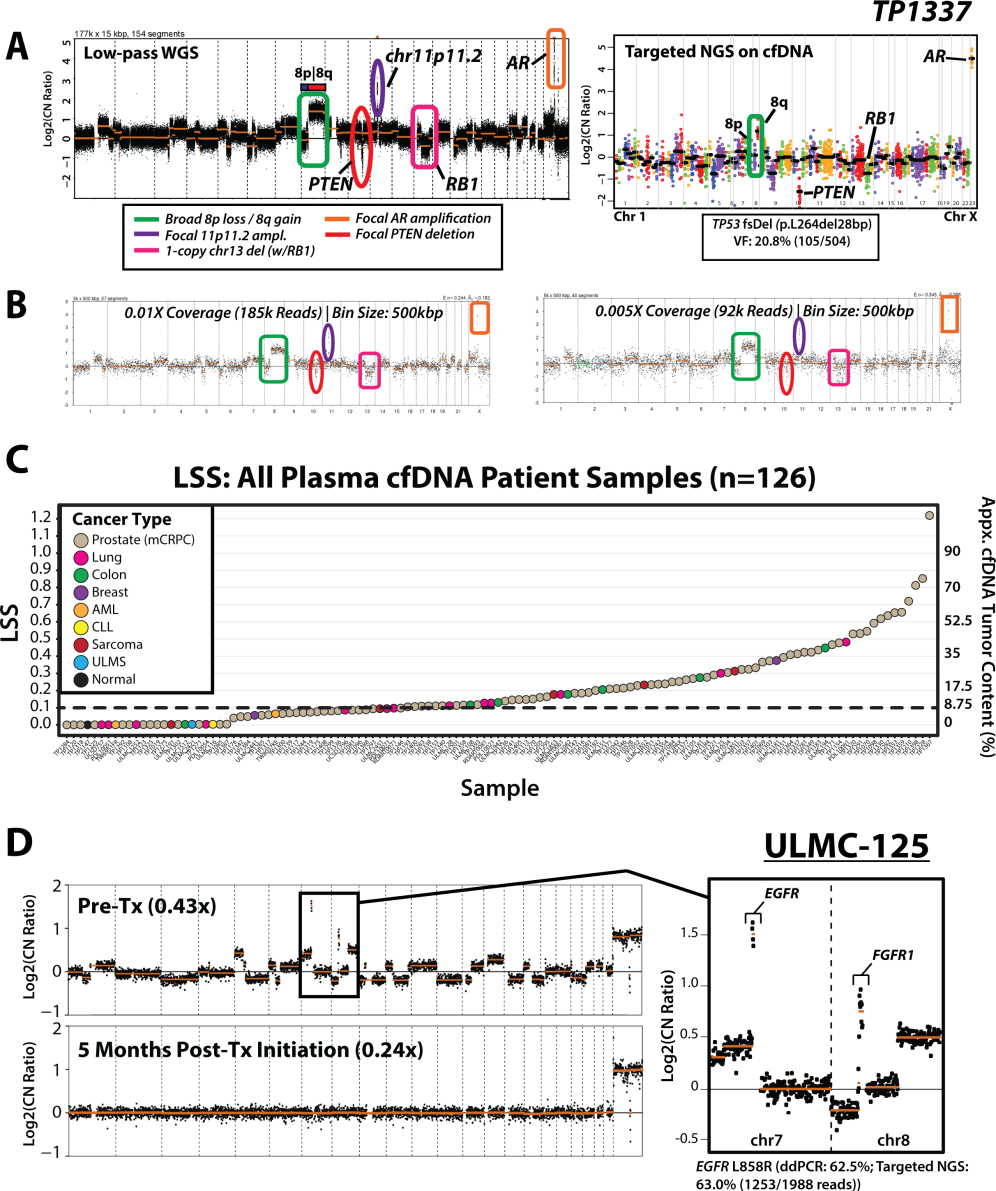
cfDNA tumor content approximation and disease monitoring applications for targeted and ultra-low-pass whole-genome sequencing (WGS) of cell-free DNA from patients with advanced cancer (**A**) Genome-wide log2(CopyNumberRatio) (Log2CN) calls for TP1337, a high tumor content cfDNA sample from a patient with mCRPC, are displayed for low-pass WGS data (0.82x whole-genome coverage) and targeted NGS data. Key copy-number alterations (CNA) detected are circled, including broad gain of 8q (green), focal amplification of chr11p11.2 (purple) and *AR* (orange), and focal *RB1* (1-copy; pink) and *PTEN* (2-copy; red) deletions. Copy number and mutation data from deep coverage targeted NGS data is provided at right from unamplified TP1337 cfDNA (1,102× targeted NGS coverage) using the DNA component of the Oncomine Comprehensive Assay (OCP), a pan cancer NGS panel developed for FFPE tissue samples. For genes with sufficient amplicons for CNA calling, amplicon (dots) and gene (black bars)-level log base 2 copy number ratio (Log2 [CN Ratio]) estimates (compared to a composite reference sample) are plotted. All CNAs seen by low-pass WGS are detected via targeted NGS CNA analysis (chr11p11.2 is not targeted by OCP). A prioritized high confidence somatic 28 bp frameshift deletion in *TP53* (p.L264del28bp; variant fraction (VF) = 20.8% (105/504 total sequencing reads)) detected by OCP is shown in the inset box. (**B**) *In silico* downsampling experiments highlight the ability to detect both focal and broad copy-number alterations from TP1337 cfDNA WGS data at whole-genome coverages down to 0.005×. Bin size and number of high-quality (MAPQ ≥ 37) mapped reads used for copy-number analysis are indicated at each coverage, and regions affected by copy-number alterations detected in original low-pass WGS are circled. (**C**) Distribution of cfDNA tumor content estimates (right axis) from least-squares distance metric (LSS) values (left axis) for 124 patient cfDNA samples (123 from patients with advanced cancer, including TP1178 (from a patient with untreated advanced prostate cancer), along with 1 normal control sample (TP1147). All patient samples are colored by cancer type (indicated in the legend). (**D**) Low-pass WGS copy-number for pre- and post- EGFR inhibitor (erlotonib) treatment plasma cfDNA samples from ULMC-125, a patient with metastatic lung cancer. Multiple whole-chromosome and arm-level gains/losses as well as focal amplifications are present in the pre-treatment cfDNA sample with high tumor content. A zoomed view of chromosomes 7 and 8 show focal *EGFR* and *FGFR1* amplifications in the pre-treatment sample (an activating *EGFR* L858R mutation was previously detected at 62.5% variant fraction by digital droplet PCR [ddPCR]). Low-pass WGS sequencing of a cfDNA sample taken 5 months post-treatment initiation (bottom) showed no detectable copy-number alterations genome-wide, and no detectable L858R mutation by ddPCR analysis (L858R variant fraction: 0.0%). Low-pass WGS copy-number bin size: 500 kbp; segmentation *p*-value threshold: 0.01.

Subsequent *in silico* dilution and downsampling experiments of cell line (sheared gDNA) and patient cfDNA WGS data facilitated development of a heuristic tumor content approximation metric (least squares statistic; LSS), while highlighting our ability to recapitulate both broad and focal copy-number alterations across tumor contents as low as 5% (see Supplementary Figures 3–5, Supplementary Methods). While detection of focal amplifications by low-pass cfDNA WGS is also dependent on absolute copy-number of amplified gene(s) in the tumor, high-level focal amplifications (>4 copies) are frequent across TCGA and advanced cancers [48, 49], and abundant and detectable in our patient cohort (described below). An illustrative example of a genome-wide copy-number profile from cfDNA collected from a patient (TP1337) with mCRPC after progression on second generation anti-androgens abiraterone and enzalutamide is shown in Figure 2A. TP1337 harbored focal *AR* amplification, chr8q gain, focal 2-copy *PTEN* loss, and one-copy loss on chr13 including *RB1*, representing the majority of the most common CNAs in mCRPC [45]. Figure 2B further displays the ability of our approach to detect both broad and focal CNAs down to 0.005x (~82,000 reads) in TP1337, with routine robust detection of focal amplifications in cell lines and high tumor content mCRPC samples at 0.01x coverage (Supplementary Figures 6 and 7). While ultra-low-pass (0.005×) is expected to have greatest clinical utility in high-tumor content cfDNA samples, these results support the fidelity of copy number profiling from cfDNA using our low-pass WGS based PRINCe approach and the capacity to leverage this workflow to both approximate tumor content and identify high level focal amplifications, a key therapeutic class of somatic alterations in cancer.

### Application of PRINCe to patient cfDNA sample cohorts and utility in disease monitoring

To demonstrate feasibility and utility of PRINCe in representative clinical scenarios, we next assessed cfDNA from two patient cohorts, one comprised of 31 samples from 24 individual patients with metastatic colorectal, breast, or lung cancers, uterine leiomyosarcoma, sarcoma, or leukemia, and another comprised of 93 samples from 75 patients with mCRPC (including patients with both low and high volume disease) (Supplementary Table 1). Across the 124 total patient samples, 74 (59%) had LSS values ≥ 0.1, and thus an estimated cfDNA tumor content of > 8.75% (Figure 2C, Supplementary Results). PRINCe enabled routine detection of actionable focal copy-number alterations (including focal *EGFR* and *FGFR1* amplifications) across patient samples in our non-mCRPC cohort (Figure 2D); combining this approach with targeted cfDNA enabled robust detection of ddPCR validated informative point mutations or indels (including *EGFR* exon 19 deletions) (see Supplementary Results). PRINCe profiling of serial cfDNA samples from several patients highlighted utility in evaluating treatment response, disease monitoring, and identification of candidate biomarkers of treatment response in a patient (PD-L1006_1) with stage IV lung adenocarcinoma who achieved a complete response to PD-L1 checkpoint inhibition immunotherapy (see Supplementary Results). While there remains clear utility in specific contexts for profiling disease recurrence at extremely low tumor content using high-depth, ultra-sensitive or personalized sequencing/ddPCR methodologies [32, 50, 51], our results suggest substantial potential clinical utility across cancer types from low-cost identification of pre-treatment genome wide CNA profiles and cfDNA tumor content estimates via highly scalable whole-genome and targeted cfDNA NGS-based profiling strategies to monitor disease burden and molecular evidence of response.

### PRINCe applied to metastatic castration resistant prostate cancer (mCRPC)

Given the potential impact of CNA detection in cfDNA—particularly *AR* amplification—on therapeutic decision-making in prostate cancer [23, 52, 53], we next focused on the 76 patients with mCRPC. All patients had progressive disease after androgen deprivation therapy, and the clinical characteristics are shown in Supplementary Tables 2 and 3. PRINCe was carried out on 5 normal male and 93 mCPRC patient samples (including one technical replicate, TP1052B) to average whole-genome coverage of 0.32x (range: 0.02–1.30x)). Of 93 mCRPC cfDNA samples, 60 (65%) had estimated tumor contents greater than 8.75% by LSS analysis (LSS ≥ 0.1), our minimum threshold for accurately estimating tumor content, and were considered as high tumor content. Low-pass WGS of one cfDNA sample (TP1330) identified a single 19Mb deletion on chr20 (20q11.21–20q13.2) leading to elevated LSS, while by targeted NGS this sample also carried a *U2AF1* S34F COSMIC hotspot mutation (variant fraction = 30%, 527 covering reads; Supplementary Table 4), consistent with contaminating white blood cell cfDNA in the presence of concurrent myelodysplastic syndrome [54], and thus this sample was considered as low tumor content for subsequent analyses (Figure 2B and Supplementary Figure 8; see Methods). In total, the 63% (59 of 93) of mCRPC samples with estimated tumor content >8.75% represent a similar proportion of mCRPC samples to that reported as having sufficient tumor derived cfDNA for array CGH and targeted NGS based assessment described by Wyatt et al. [55].

Unsurprisingly, 68 of 93 mCRPC cfDNA samples (73%) showed evidence of detectable chromosome 8p losses and/or 8q gain (known early alterations in prostate carcinoma progression [56, 57]), including 58 of 59 (98%) high tumor content samples (Supplementary Figure 9). In total, 14 of 93 (15%) mCRPC cfDNA samples also demonstrated detectable segmented 21q22.2 copy-number deletions consistent with deletion leading to *TMPRSS2:ERG* gene fusion, another known early event in prostate oncogenesis [58, 59] (Supplementary Figure 10). Focal copy number alterations were also frequent, including *PTEN* deletion (20 of 59 (28.8%) high tumor content cfDNA samples, 11 (65%) of which are focal deep deletions (Supplementary Figure 10)), and focal *AR* amplification (36 of 93 (39%) cfDNA samples, including 32 of 59 (54%) high tumor content mCRPC samples) (Supplementary Figure 10, Supplementary Table 1), both of which are biomarkers of poor prognosis and/or resistance to second-line anti-androgens (abiraterone and enzalutamide), particularly when observed in cfDNA [23, 52, 53, 60] (see Supplementary Results). Focal *RB1* deletion, a frequent alteration in neuroendocrine/small-cell prostatic carcinoma [45, 61], was also detectable by our approach, with 4 samples (4.3%) (4 patients) exhibiting focal deep deletions (Supplementary Figure 10), including 1 from a patient (TP1320) with detectable *AR* amplification, who (post-ADT and a single course of docetaxel) progressed rapidly on abiraterone over the course of 3 months on therapy with PCa-related death 4 months after cfDNA profiling (Supplementary Table 2; see Supplementary Results).

Notably, PRINCe assessment of cfDNA sample TP1291 paired with targeted NGS of the matched unamplified cfDNA (described below) identified a broad 1-copy copy-number loss affecting *BRCA2* and *RB1* in combination with a Clinvar pathogenic *BRCA2* germline R2494X stop-gain SNV at a variant fraction (71%, 1,022 variant-containing reads) consistent with copy-number deletion of the non-mutated copy of the gene and biallelic inactivation of *BRCA2* (Supplementary Figure 11). Prior to cfDNA sample collection, the corresponding patient progressed rapidly through courses of abiraterone, enzalutamide, docetaxel, and cabazitaxel over the 11 months prior to cfDNA sample collection, consistent with known poor prognosis for *BRCA*-mutant men with prostate cancer [62], confirming important utility for cfDNA profiling in guiding PARP inhibitor treatment in patients with advanced prostate cancer [30, 31]. Additional PRINCe assessments detected a putative complex rearrangement affecting *BRCA1* in a patient with mCRPC, along with clinically relevant copy-number alterations in advanced treatment-naïve patients with heavy tumor burden (Supplementary Figure 11; see Supplementary Results). Overall, these results highlight our capacity to detect therapeutically relevant focal copy-number deletions from low-pass cfDNA WGS in patients with mCRPC and support potential clinical utility in informing precision oncology workflows for patients with advanced prostate cancer.

### PRINCe to guide additional precision oncology testing

In the absence of immediately actionable copy-number alterations by low-pass WGS, a priori tumor content approximation from low-pass cfDNA WGS can enhance subsequent precision medicine workflows by directly informing requisite strategies or coverages needed for meaningful NGS profiling (Figure 1C). For example, we hypothesized that in patients with relatively high cfDNA tumor content (e.g. >10%), routine tumor tissue profiling NGS strategies would be sufficient to detect relevant alterations, rather than ultra-high depth, high fidelity (e.g. single molecule barcoding) sequencing as typically performed for cfDNA NGS. Hence, we subjected separate 1–20 ng aliquots of unamplified cfDNA from 61 of our patient samples (including 46 mCRPC samples, 11 high tumor content non-mCRPC samples, and 4 male control samples with sufficient DNA; see Supplementary Table 1), as well as the undiluted artificial VCaP and UMUC5 cfDNA samples as positive controls, to targeted multiplexed PCR based NGS using the DNA component of the Oncomine Cancer Assay (OCP) [4], the panel being used in the NCI sponsored MATCH trial performing NGS on tumor tissue.

Sequencing of pooled patient samples resulted in a median average coverage of 1,075x (range: 42–17,944×), with average uniformity of 96.0% (higher than typically observed for FFPE DNA samples [4]). OCP on cfDNA confirmed high level *EGFR* amplification in UMUC-5, and high level *AR* amplifications in VCaP and 23 of 23 (100%) high tumor content mCRPC samples. In TP1337 (see Figure 2A), OCP on cfDNA validated all key somatic copy-number alterations detected by low-pass cfDNA and detected a 28 bp *TP53* frameshift deletion (L264del28bp, variant frequency 20.8% with 504 covering reads) (Figure 2A). Of note, we observed high correlation between gene-level copy number alterations (absolute_value [targeted NGS log2(CopyNumberRatio)] ≥ 0.5) by targeted sequencing and low-pass WGS calls from PRINCe assessment of patient cfDNA samples (Pearson correlation coefficient: 0.92, *p* < 0.001), and *in silico* down-sampling experiments in patient and cell line cfDNA samples suggest mean coverages as low as 50× enable reliable detection of known putative clonal somatic point mutations, indels, and copy number variants in samples with high tumor content (Supplementary Figures 12 and 13). Taken together, these results underscore the potential for PRINCe followed by targeted sequencing (tuned to cfDNA tumor content) as part of a high-throughput, cost-effective clinical or translational research NGS workflow.

### PRINCe concordance with comprehensive tissue-based profiling

To assess the potential utility of PRINCe cfDNA assessment in the context of comprehensive tissue-based precision oncology workflows, we focused on 26 of the 76 men (34%) with mCRPC profiled by cfDNA low-pass WGS (corresponding to 31 of 93 (33%) mCRPC cfDNA samples) where synchronous or asynchronous comprehensive whole exome and whole transcriptome profiling was attempted on fresh frozen or FFPE biopsy tissue specimens (median number days between tissue- and cfDNA specimen collection: 137 (range: 0–682 days)). Of 26 men, 4 (15%) had either insufficient tumor content for comprehensive tissue profiling or incomplete tissue profiling data for analysis. Notably, all 4 men had cfDNA samples that yielded clinically informative results, including 4/4 (100%) with detectable focal *AR* amplification, while 4 of 5 patient-matched cfDNA samples were taken pre-biopsy highlighting important opportunities for optimized resource allocation in precision medicine workflows (see Supplementary Results). Collectively, this supports complementary clinical utility for plasma cfDNA profiling when paired with comprehensive tissue-based NGS workflows as a first-stage “screening” strategy.

**Figure 3:**
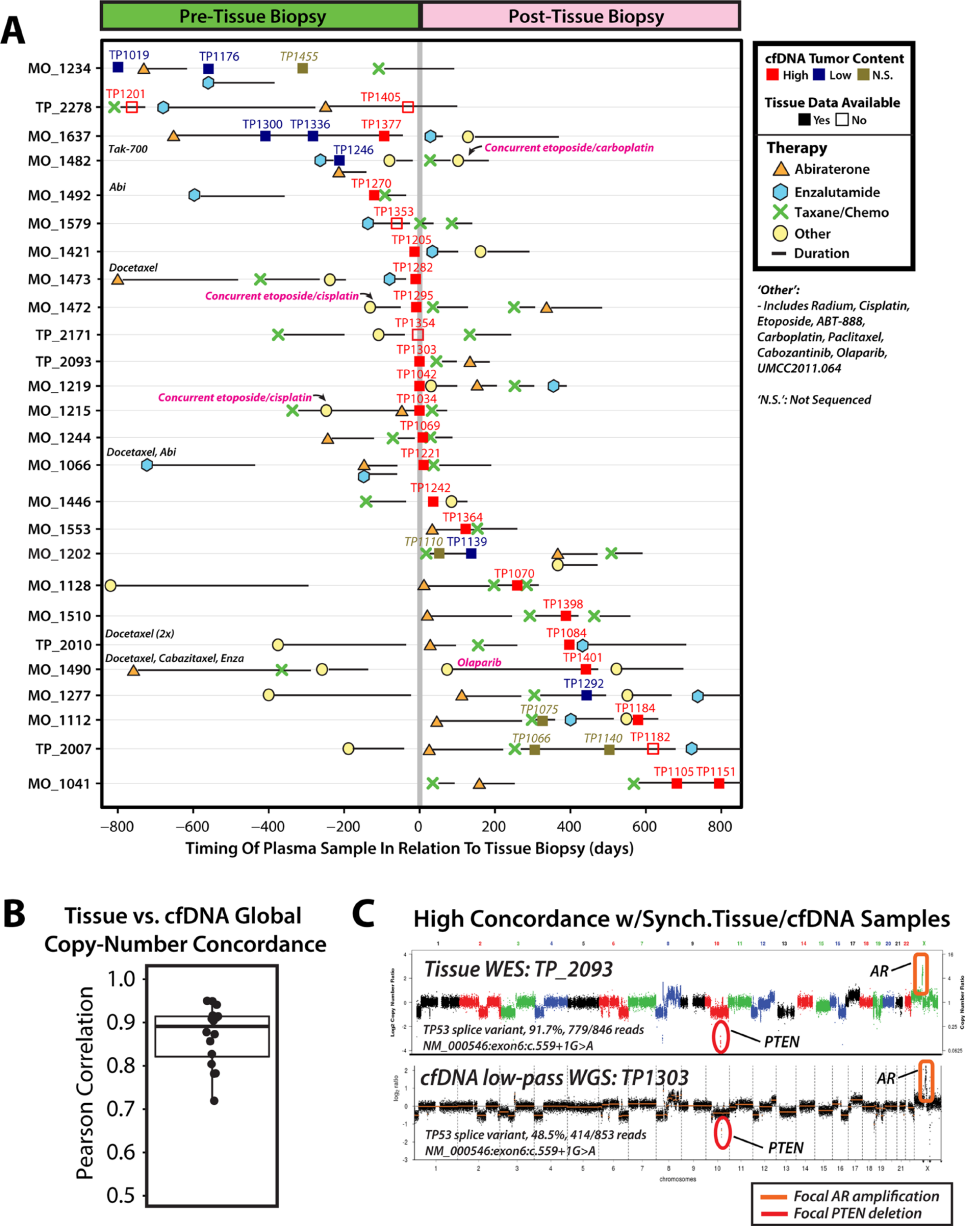
Comparison of synchronous and asynchronous tissue and cfDNA biospecimens collected from patients with metastatic castration-resistant prostate cancer (mCRPC) yields highly concordant genome-wide copy number profiles (**A**) Treatment and cfDNA sample collection timeline plotted in relation to tissue specimen collection date for 26 men with metastatic castration-resistant prostate cancer (mCRPC) eligible for tissue-based comprehensive whole-exome and whole-transcriptome NGS profiling. Treatment start and cfDNA sample dates are plotted relative to tissue specimen collection date (denoted by solid vertical gray line) for each individual. As indicated in the legend, treatments have been divided into 4 separate categories, including: abiraterone (orange triangle), enzalutamide (blue hexagon), taxane-based chemotherapy (green ‘X’) and other (yellow circle), and treatment duration is indicated by solid black horizontal lines extending rightward from treatment start dates. Therapies categorized as ‘other’ include: radium, cisplatin, etoposide, ABT-888, carboplatin, paclitaxel, cabozantinib, olaparib, and UMCC2011.064. Where appropriate, ‘other’ treatment including etoposide and cisplatin or carboplatin for individuals with prostate cancer containing small cell/neuroendocrine features are noted. As indicated in the legend, samples are colored by LSS-based tumor content approximation with high (LSS > 0.1, red), low (LSS < 0.1, blue), and not sequenced (‘N.S.’, brown). For a subset of men, tissue-based molecular data was not available, as indicated by filled (tissue data available) or unfilled (tissue data not available) squares. Displayed sample dates are restricted to +/− 800 days from date of tissue specimen collection, and therapies administered > 800 days before tissue specimen collection are written at the left-hand side of corresponding individual timelines. (**B**) Correlations between genome-wide tissue and cfDNA segmented copy-number profiles are plotted for 16 patients with available comprehensive tissue NGS profiling data and PRINCe assessment of ≥ 1 high tumor content cfDNA sample (see Methods). Each point represents the correlation of genome-wide copy number profile for a single cfDNA sample as compared to the patient-matched tissue-based copy-number profile. A box-and-whisker plot behind points indicates the interquartile range (IQR), with the top and bottom of box representing 25th and 75th percentile, respectively, while bold horizontal line within the box represents the median correlation value. Whiskers stretch to 1.5 times the IQR for this sample distribution. (**C**) Tissue whole exome sequencing (WES) (top; tissue id: TP_2093) and cfDNA low-pass whole genome sequencing (WGS) (bottom; cfDNA id: TP1303) genome-wide copy-number profiles for biospecimens collected on the same day from a patient with mCRPC (TP_2093). Genome-wide copy-number concordance is statistically significant (Pearson correlation coefficient: 0.94, *p* < 0.001), and focal 2-copy deletion of PTEN and focal high-level AR amplification are cleared detected in both the tissue and cfDNA as indicated. A TP53 splice variant (NM_000546:exon 6:c.559+1G>A) identified via WES tissue profiling (91.7% variant fraction (VF), 846 covering reads) is also detected by cfDNA targeted NGS (48.5% VF, 853 covering reads).

Global copy number concordance across tissue and cfDNA profiling has been poorly explored in mCRPC and other cancers. Hence, we next assessed the 22 men with comprehensive tissue-based profiling and at least 1 profiled cfDNA sample (range of cfDNA samples per individual: 1–3), of which 18 (82%) had a cfDNA sample w/high cfDNA tumor content amenable to analysis (Figure 3A, Supplementary Table 2). Despite variable specimen tumor content and sample synchronicity, genome-wide segmented tissue-based copy-number profiles were highly correlated (median *r* = 0.87 [range: 0.54–0.95]; Figure 3B) with whole genome cfDNA segmented copy-number profiles for the 16 of 18 (89%) individuals with fresh frozen tissue specimens, and this concordance was not significantly associated with time between cfDNA and tissue specimen collection (*p* = 0.72, two sample *t*-test) (Supplementary Figure 14, Supplementary Table 3, Supplementary Methods). For 6 of 18 men (33%) with high tumor content cfDNA samples and tissue-based profiles, clear 21q22.2 copy-number deletions (consistent with TMPRSS2:ERG gene fusion) detected by cfDNA WGS was also detected in tissue-based DNA profiling, with *TMPRSS2:ERG* fusion isoform expression confirmed by tissue-based RNAseq in 5 of 6 men (Supplementary Figure 10, Supplementary Table 1). Of 18 men with tissue profiling data, 12 (67%) harbored focal *AR* amplifications and 11 of 12 (92%) patient-matched high tumor content cfDNA samples show concordant detectable *AR* amplifications (example in Figure 3C; Supplementary Table 3). By targeted NGS of patient-matched cfDNA samples, 24/28 (86%) somatic point mutations and indels present in tissue specimens at variant fractions ≥ 10% targeted by our panel were detected in cfDNA samples, including 20/21 (95%) in matched high tumor content cfDNA samples and 15/15 (100%) in high tumor content cfDNA samples collected ≤ 200 days from tissue collection (Supplementary Figure 15, Supplementary Tables 4 and 5). Collectively, these results suggest PRINCe assessment of routine cfDNA samples from men in mCRPC may enable highly scalable, robust identification of putative clonal somatic alterations consistent with comprehensive profiling results from synchronous tissue samples.

**Figure 4:**
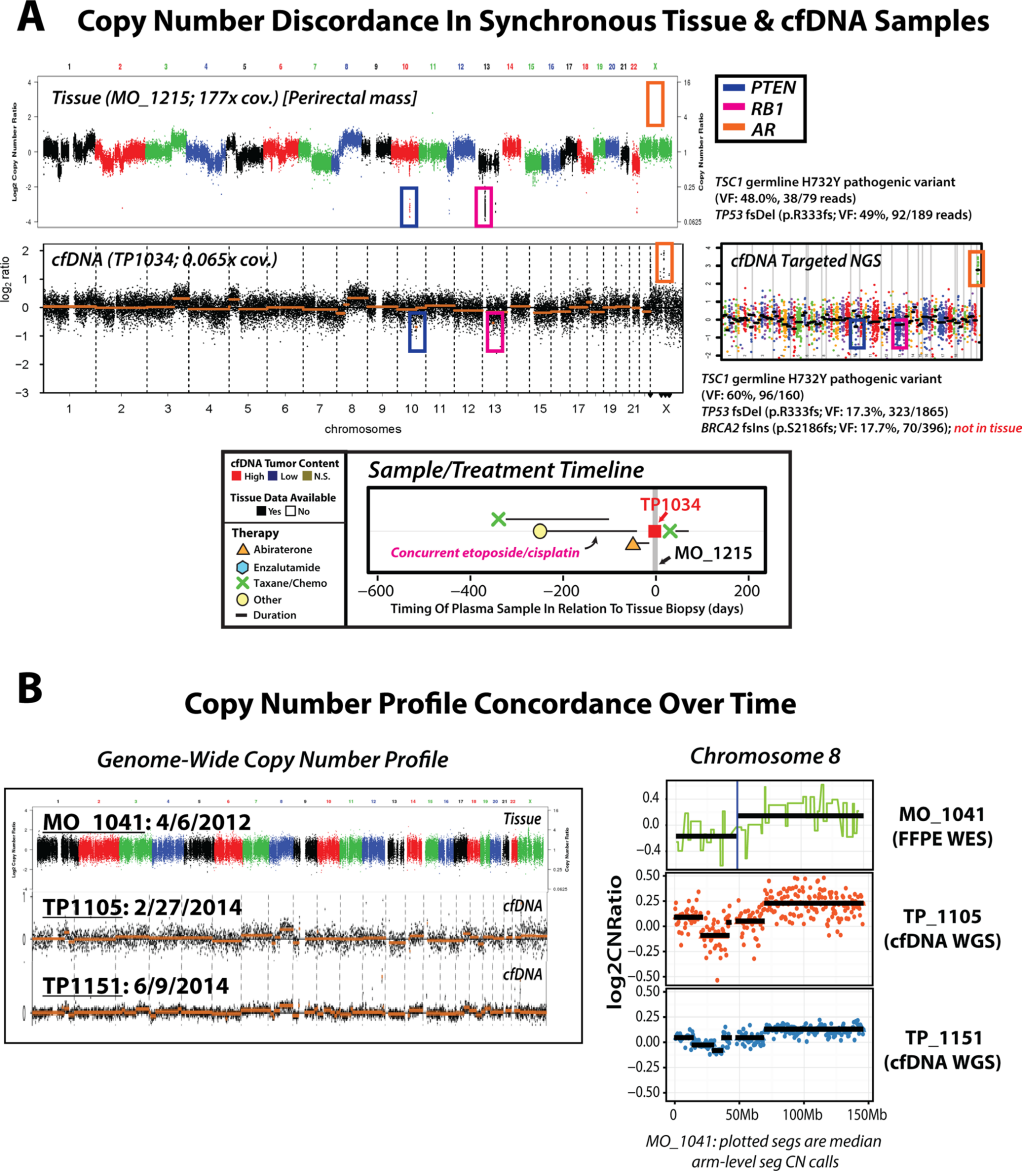
Unique precision oncology considerations identified via serial and synchronous tissue and cfDNA NGS-based profiling in patients with advanced prostate cancer (**A**) Genome-wide (tissue and cfDNA) and targeted (cfDNA only) NGS copy number profiles are displayed, along with treatment and sample timeline, for synchronous (same-day) tissue and cfDNA specimens from a patient with metastatic castration-resistant prostate cancer (mCRPC) with a history of both primary prostatic adenocarcinoma and a metastatic lesion with small cell carcinoma/neuroendocrine features. Tissue whole exome sequencing (WES) copy number analysis of a frozen perirectal mass tissue biopsy specimen (top left) revealed focal, deep deletions in both *PTEN* and *RB1*, and no *AR* copy-number alterations, consistent with histological reports of high-grade poorly differentiated carcinoma with neuroendocrine features. Individual dots in tissue WES copy-number profile represent exon-level copy-number estimates displayed in genome order, and dots are colored by corresponding chromosomes. cfDNA low-pass whole genome sequencing (WGS) copy number profiling identified focal deep deletions in *PTEN* and *RB1*, as well as high level focal *AR* amplification, highlighting circulating evidence of both *AR*-driven and *AR*-independent clones. Individual dots in cfDNA low-pass WGS plot represent bin-level copy-number estimates displayed in genome order (left to right), with segmented copy-number alterations represented by orange horizontal lines. Both tissue (WES) and cfDNA (targeted NGS; copy-number prfile ) mutation profiling identified a *TSC1* germline H732Y pathogenic variant (tissue: 48% variant fraction (VF), 79 covering reads; cfDNA: 60% VF, 160 covering reads) and somatic *TP53* frameshift deletion (p.R333fs; tissue: 49% VF, 189 covering reads; cfDNA: 17%, 1865 covering reads), while cfDNA targeted NGS identified a *BRCA2* frameshift insertion (p.S2186fs; 18% VF, 396 covering reads) not present in the tissue sample, further supporting detection of multiple clones via cfDNA PRINCe assessment. The cfDNA targeted NGS copy number profile is presented at right, showing confirmation of focal *PTEN* and *RB1* deletions along with high-level focal *AR* amplification as seen by low-pass WGS. Zoomed view of treatment and sample timeline for this patient is presented at bottom, as previously described (see Figure 3). (**B**) Genome-wide (left) and chromosome 8 (right) copy number profiles from multiple biospecimens taken over time from a single patient with metastatic castration-resistant prosate cancer (mCRPC) (tissue id: MO_1041; cfDNA ids: TP1105 and TP1151). WES of a formalin fixed paraffin embedded (FFPE) tissue biopsy specimen (top left) revealed low but detectable tumor content, and identified copy-number loss affecting 8p and arm-level gain of 8q (at right). Low-pass WGS of a cfDNA specimen collected almost 2 years after tissue biopsy (TP1105, middle left) revealed elevated cfDNA tumor content with frequent copy-number alterations genome-wide, including copy-number loss affecting chr8p and arm-level gain of chr8q (displayed at right), as detected in initial tissue profiling. A subsequent cfDNA sample (TP1151) again showed detection of elevated cfDNA tumor content and a highly concordant genome-wide copy number profile, with faithful representation of the 8p loss and 8q gain events detected in previous specimens. Overall, these results highlight the consistent representation of early genomic events as inferred from circulating tumor DNA profiled in our cohort.

Clinically relevant discrepancies between synchronous cfDNA and tissue profiles, however, were also identified. In one patient with a history of both primary prostatic adenocarcinoma and a metastatic lesion with small cell carcinoma/neuroendocrine features (TP1034/MO_1215), PRINCe assessment of synchronous (same-day) specimens detected a clear focal *AR* amplification in the cfDNA that was absent in the tissue based profiling of a prostatic neuroendocrine/small cell carcinoma focus (despite identical prioritized somatic point mutations), consistent with circulating evidence of both *AR*-driven and *AR*-independent clones (Figure 4A). Further, while previous reports suggest cfDNA clonal representation of known early copy-number events (including chr8p/8q changes) in men with mCRPC may vary over time and therapy [24, 40], analyses in our cohort reveal stable representation of early genomic events in tissue and serial patient-matched plasma cfDNA samples (Figure 4B, Supplementary Results). Overall, these results suggest noninvasive profiling may yield high concordance with near-synchronous tissue profiling for clinically relevant molecular alterations, and may provide unique complementary advantages and opportunities for expansion into treatment-naïve patient cohorts.

### Evaluating prognostic utility of cfDNA biomarkers

**Figure 5:**
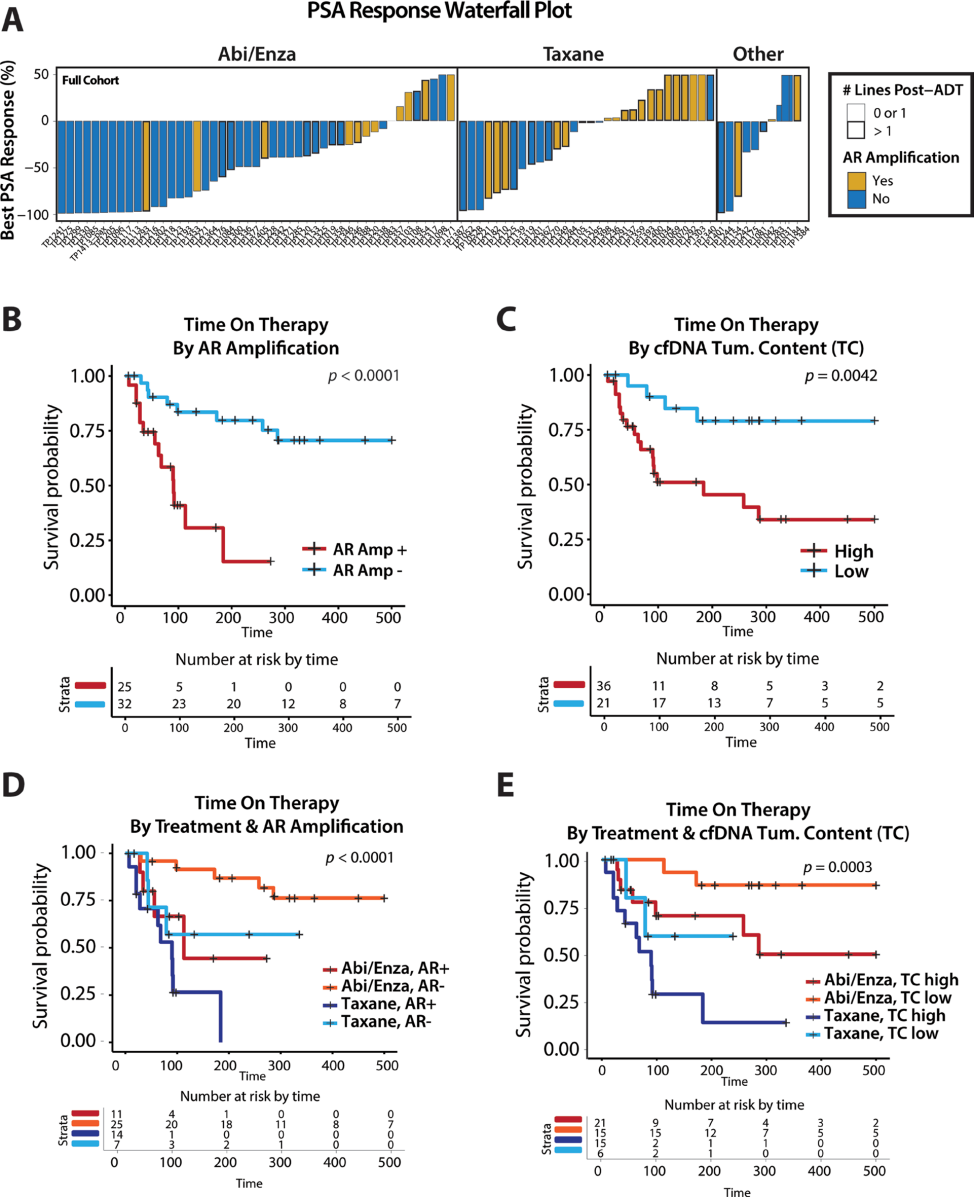
Exploratory analyses of association between circulating biomarkers and outcome in patients with metastatic castration-resistant prostate cancer (mCRPC) supports cfDNA detectable AR amplification as a poor overall prognostic factor independent of treatment type (**A**) Waterfall plot summarizing prostate specific antibody (PSA) response for all samples from men with mCRPC with complete PSA data (*n* = 90). Height of bars represent the percentage change in PSA response as calculated by subtracting the PSA level at sample date from the best PSA observed after sample date while on the current or initiated treatment, and dividing by starting PSA value. Bars are ordered horizontally within treatment category (Abi/Enza, Taxane, or Other) by PSA response. Bars are colored by cfDNA detectable *AR* amplification status (yellow = cfDNA detectable AR amplification; gray = no cfDNA detectable AR amplification) and bars corresponding to samples taken from men who have received more than one line of therapy post-ADT are outlined in bold. (**B**–**E**) Kaplan-Meier survival curves are plotted for analyses exploring association between cfDNA detectable *AR* amplification (B, D) or cfDNA tumor content (C, E) and total time on therapy in both unstratified (B-C) and stratified (D-E; by treatment type) analyses of our mCRPC cohort. Unstratified analysis of single cfDNA samples from men on or starting taxane-based chemotherapy or second-generation anti-androgens abiraterone or enzalutamide (*n* = 57 men) highlight significant differences in time on therapy for both (B) cfDNA detectable *AR* amplification (Kaplan-Meier log-rank test, chi-square = 15.3, *p* < 0.0001) and (C) elevated cfDNA tumor content (Kaplan-Meier log-rank test, chi-square = 8.2, *p* < 0.0042). Analyses stratified by treatment (starting or on taxane vs. abiraterone/enzalutamide) show (D) cfDNA detectable *AR* amplification (yes/no) (Kaplan-Meier log-rank test, chi-square = 21.9, *p* < 0.0001) and (E) cfDNA tumor content (Kaplan-Meier log-rank test, chi-square = 18.9, *p* = 0.0003) again demonstrate significant differences in time on therapy. Survival curves are colored by corresponding strata, and risk tables at selected timepoints are displayed below each Kaplan-Meier plot.

cfDNA detectable *AR* amplification has been reported as a biomarker predicting therapeutic resistance to second generation anti-androgens (abiraterone/enzalutamide) in several studies [23, 52, 53], while circulating tumor cell (CTC) detectable ligand independent *AR* splice variant (*AR-V7*) has been reported as predictive of abiraterone/enzalutamide resistance *and* taxane chemotherapy sensitivity [63, 64]. While our mCRPC cohort was not designed specifically to assess associations between circulating biomarkers and clinical outcome or therapeutic response, our cohort contained a large number of men on—or starting—second generation anti-androgens, as well taxane based chemotherapies. In exploratory analyses in our full cohort, we observed an enrichment of cfDNA detectable *AR* amplification in samples from patients with limited PSA response (Figure 5A), with both cfDNA detectable *AR* amplification (Kaplan-Meier log-rank test, chi-square = 15.3, *p* < 0.0001; Figure 5B) and elevated cfDNA tumor content (Kaplan-Meier log-rank test, chi-square = 8.2, *p* < 0.0042; Figure 5C) showing a significant association with reduced time on therapy. Further, stratifying by therapy (starting or on taxane vs. abiraterone/enzalutamide), we see that both *AR* amplification (yes/no) (Kaplan-Meier log-rank test, chi-square = 21.9, *p*<0.0001; Figure 5D) or cfDNA tumor content (Kaplan-Meier log-rank test, chi-square = 18.9, *p* = 0.0003; Figure 5E) again show significant differences in time on therapy, suggesting cfDNA detectable *AR* amplification (and high cfDNA tumor content) may be a potentially prognostic marker for resistance to both second generation anti-androgen therapy and taxane chemotherapies. These results are consistent with those seen when restricting analyses to samples from patients on or starting therapy separately (Supplementary Figure 16), and together confirm previous reports that cfDNA detectable *AR* amplification predicts resistance to abiraterone or enzalutamide [23, 52, 53], while supporting *AR* amplification (and high tumor content) as a more general poor prognostic factor, similar to circulating tumor cell (CTC) count [65, 66].

## DISCUSSION

Many comprehensive precision oncology NGS approaches carry up-front coverage and sequencing requirements (aimed at maximizing sensitivity and specificity) that limit clinical implementation across cancer types in the current era of limited reimbursement, particularly using cfDNA (where estimated tumor content can be < 0.01% in early stage disease [27]). Given current precision oncology NGS testing is typically performed in patients with multiple-therapy refractory advanced cancers usually exhibiting significant disease burden [67], here we describe a pan-cancer, rapid, inexpensive, ultra-low pass NGS cfDNA (PRINCe) based precision oncology first stage “screening” approach. Our approach can 1) direct therapy in patients with actionable CNAs, 2) guide precision oncology workflows based on cfDNA tumor content approximation in the absence of actionable CNAs, and 3) identify genome wide CNA profiles that can be used for treatment monitoring. We show this highly scalable approach cfDNA WGS approach can be deployed at effective whole-genome coverages down to 0.01x from as little as 10pg of DNA, and that it facilitates robust detection of clinically relevant CNAs and tumor content approximation in samples with tumor contents >~10%, suggesting substantial utility as a high-throughput, cost-effective screening tool in research and clinical laboratories (with appropriate validation).

As CNAs may not be informative in all cancers, and many patients may have insufficient tumor content to identify high level CNAs, results from our approach can be used to guide additional precision oncology NGS profiling of the same cfDNA sample or fresh frozen or archived FFPE tissue-based NGS profiling, with sequencing approach and coverage tuned to tumor content. Supporting this tiered approach, we performed targeted multiplexed PCR based NGS on residual unamplified cfDNA from 61 cfDNA samples from patients with advanced cancer, confirming focal amplifications and identifying potentially informative mutations and indels at high concordance with known putative clonal alterations (25/26, 96%) in cfDNA samples with high tumor content. Comparisons between cfDNA and comprehensive tissue-based profiling in a subset of patients highlight substantial concordance for both somatic mutational and copy-number profiles, while elucidating important potentially complementary utility for cfDNA-based profiling strategies.

Limitations of our approach include the need for multiple assays, particularly in tumor types with few CNAs or where chromosomal rearrangements must be assessed. Likewise, in clinical scenarios where cfDNA tumor content is expected to be very low, up front ultra-deep cfDNA sequencing or ddPCR (as currently performed) is more appropriate, though our ability to detect known early broad copy-number events (e.g., 8p loss, 8q gain) in prostate carcinoma progression at low cfDNA tumor content (see Supplementary Figure 9) suggests potential expanded utility of our approach at lower tumor contents than currently implemented (paired with more comprehensive approaches when necessary). Further refinement of our tumor content approximation approach (see Supplementary Methods) through assessment of informative heterozygous SNPs or incorporation of a matched normal genomic DNA would enhance the precision and lower limits of our tumor-derived cfDNA fraction estimates, though costs and feasibility in a clinical sequencing workflow are key considerations. While PRINCe is necessarily limited to megabase resolution for copy number alteration detection at ultra-low-pass (~0.01x) whole-genome coverage, smaller (multi-kilobase) clinically relevant focal alterations (including focal *PTEN* deletion) can clearly be detected at 0.01–0.1x genome-wide coverages with sufficient cfDNA tumor content (Figure 5B). Importantly, our approach can be routinely completed in 2–3 days and when performed at 50% capacity on an Ion Torrent Proton sequencer (currently limited by Ion Torrent barcodes incorporated in ThruPLEX library construction), 96 samples could be sequenced per single Ion Torrent Proton P1 chip at list reagent costs of ~$70 per sample for library construction and NGS. Taken together, these observations suggest the proposed workflow may be amenable to high volume, cost-effective ultra-low-pass WGS screening protocols.

Applied to a large mCRPC cohort, our approach showed high overall concordance between our cfDNA genome-wide CNA profiles with tissue-based profiles derived from whole exome sequencing in a precision medicine program [45]. In addition, we demonstrated that cfDNA detectable *AR* amplification not only predicts poor response to second generation anti-androgens, consistent with other published reports [23, 53], but it also portends poor prognosis for patients treated with taxane based chemotherapy. Hence, cfDNA detectable *AR* amplification may be a more general poor prognostic factor, unlike *AR-v7*, which has been reported to confer resistance to anti-androgens and sensitivity to taxanes [63, 64]. An important limitation of these results is that this was not assessed in the context of a clinical trial, and men in our study treated with taxanes were more advanced and had been treated with more lines of therapy post-ADT. Hence, prospective confirmation of our findings will be required.

In summary, we have demonstrated the feasibility and potential utility of PRINCe, a broadly applicable, rapid, inexpensive cfDNA WGS screening assay for precision oncology that can robustly detect clinically informative CNAs from cfDNA at low tumor content using effective whole-genome coverage as low as 0.01×. This screen, while most informative in those patients with actionable CNAs and tumor content > 10%, can nevertheless be used to guide additional testing in all patients based on cfDNA tumor content approximation. Our approach highlights important potential clinical utility when paired with targeted cfDNA NGS and/or tissue-based workflows, and demonstrates unique possibilities for inexpensive disease monitoring. More generally, our study supports the potential utility of tiered approaches in precision oncology, rather than using costlier front-line approaches defined by performance necessary in the extremes.

## MATERIALS AND METHODS

### TCGA data analysis

TCGA pan-cancer copy number analyses were run on somatic segmented Affymetrix SNP6 array-based copy-number calls for 11,576 tumor samples across 32 tumor types contained in the January 28, 2016 TCGA GDAC Firehose standard data run (stddata__2016_01_28) [68] (see Supplementary Methods).

### Cell-free DNA extraction

Five milliliters of peripheral blood were collected for 93 samples from 76 patients with mCRPC and 10 healthy controls (5 male, 5 female) using K2 EDTA blood collection tubes (Cat: 366643, BD, NJ) (Supplementary Table 1), and cfDNA was isolated as described (see Supplementary Methods). For 31 samples from 24 patients with other advanced cancers (Supplementary Table 1), 10 mL peripheral blood was collected using Streck Cell-Free DNA BCT tube (Streck; NE) and cfDNA was isolated as detailed (see Supplementary Methods).

### VCaP and UMUC-5 *In vitro* dilution

We carried out *in vitro* dilution experiments using serially diluted genomic DNA from 1) VCaP cells (metastatic prostate cancer cell line) with normal male human cell-free genomic DNA at 50%, 25%, 10%, 5%, 1% and 0% dilutions, and 2) UMUC-5 cells (urothelial cancer cell line) with normal male human cell-free genomic DNA at 50%, 10%, 5%, 0% dilutions. Cell line DNA was fragmented to approximately 180 bp by Covaris AFA (Woburn, MA) focused ultrasonication. Library preparation and sequencing from undiluted and serial dilution samples was performed as for patient samples described below.

### ThruPLEX library preparation

Whole genome amplified (WGA) libraries were prepared from either cell-free DNA (cfDNA) isolated from plasma samples (median of 2.9 ng cfDNA, interquartile range [IQR] 1.73–5.79 ng, see Supplementary Table 1) or Covaris-sheared and size selected (~ 180 bp size) VCaP (1.9 ng) or UMUC-5 (2.0 ng) genomic DNA (gDNA) using the ThruPLEX RGP-0003 prototype (Takara Bio USA; Ann Arbor, MI) according to the manufacturer's protocol. Libraries were quantified using Ion Library Quantification kit by qPCR, and sequenced with 2–16 samples per Proton PI chip on an Ion Proton sequencer (Ion Torrent, Carlsbad, CA) according to the manufacturer's instructions.

### Low-pass WGS and copy-number detection

Sequencing alignment and coverage analyses were performed using Torrent Suite version 5.0.2 (Ion Torrent, Carlsbad, CA). Genome-wide copy number alterations were first called from aligned, non-PCR-duplicate reads using the QDNASeq R package (version 1.6.1) [69]. Segmented copy-number events were identified using bin-level corrected, median- and control-normalized read counts using the circular binary segmentation algorithm implemented by the DNACopy (1.44.0) R package, and final segment- and bin-level copy-number values were used for subsequent analyses as described (see Supplementary Methods). Focal CNAs were defined as CNAs 1.5–20 Mb long with a log2(CopyNumberRatio) ≥ 0.2.

### Targeted sequencing: oncomine comprehensive assay (OCP)

For 61 patient cfDNA samples (see Supplementary Table 1) and both sheared UMUC-5 and VCaP gDNA samples, we performed targeted NGS using the DNA component of the OCP, a custom multiplexed PCR-based panel of 2,530 amplicons targeting 126 genes [4]. Library preparation, data analysis, and variant and copy-number annotation and prioritization was carried out essentially as described for each sample [4, 70–72] using validated in house pipelines (Supplementary Table 1; see Supplementary Methods).

### *In silico* experiments and tumor content approximation

To establish theoretical segment-level copy-number distributions for tumor content approximation and examine efficacy across variable effect whole-genome coverages (0.005–0.01x), we carried out serial *in silico* dilution and downsampling experiments on artificial cfDNA VCaP and UMUC-5 WGS data and patient cfDNA samples (see Supplementary Methods). Using computational experiments on *in vitro* and *in silico* VCaP and UMUC-5 cell line dilution data as described in Supplementary Methods, a heuristic least squares based distance metric (LSS) was used to approximate tumor content from whole-genome copy-number data, and guide tumor content approximation for patient samples, with low tumor content samples (LSS < 0.1) specifically scanned for focal CNAs as described (see Supplementary Methods).

### Cell line cfDNA WGS vs COSMIC array-based CN calls

To evaluate the capacity of low-pass cfDNA WGS to detect copy-number alterations across variable tumor content, segmented cfDNA WGS copy-number calls for VCaP and UMUC-5 *in vitro* dilutions were compared to publically available COSMIC and targeted NGS copy-number calls, respectively (see Supplementary Methods).

### Concordance with tissue-based whole-exome sequencing copy-number profiles

Segmented log2 copy number ratio and point mutation data from whole-exome sequencing of fresh frozen tissue specimens [10, 45] was available for 22 of 26 patients also profiled by cfDNA low-pass WGS and compared to patient-matched cfDNA WGS profiles (see Supplementary Methods).

### Clinical information

All clinical and outcome information was collected, retrieved, and analyzed from internal patient tracking databases and University of Michigan Health System (UMHS) electronic health records by IRB-approved personnel.

### Statistical analyses

All statistical analyses described were carried out in R (3.2.3).

## SUPPLEMENTARY MATERIALS FIGURES AND TABLES













## Abbreviations

cfDNA: Cell-free DNA
CNA: Copy-number alterations
CRPC: Castration-resistant prostate cancer
ddPCR: Digital droplet polymerase chain reaction
NGS: Next-generation sequencing
OCP: Oncomine Cancer Assay
SNV: Single nucleotide variant
WGS: Whole genome sequencing
