# Supplementary material for: Rapid, ultra low coverage copy number profiling of cell-free DNA as a precision oncology screening strategy
Source: Oncotarget. 2017 Sep 22;8(52):89848–66. doi: 10.18632/oncotarget.21163 (PMC5685714; doi:10.18632/oncotarget.21163)
Supplement: Supplementary file 1 [file oncotarget-08-89848-s001.pdf]

Rapid, ultra low coverage copy number profiling of cell-free DNA as a precision oncology screening strategy

SUPPLEMENTARY MATERIALS

TCGA data analysis

TCGA pan-cancer copy number analyses were run on somatic (scna_minus_germline_cnv_hg19__seg) segmented Affymetrix SNP6 array-based copy-number calls for 11,576 tumor samples across 32 tumor types contained in the most recent (01/28/2016) TCGA GDAC Firehose standard data run (stddata__2016_01_28) [1]. Data was downloaded from the TCGA GDAC Firehose repository using the firehose_get utility (v0.4.6), and the fraction of genome altered (FGA) was calculated as in cBioPortal (https://groups.google.com/forum/#!topic/cbioportal/HKLa9C9m4y4). Specifically, FGA was calculated for all tumor samples as the total number of bases in regions affected by copy-number alterations with log2 (CopyNumberRatio) > 0.2 or < −0.2 divided by 3 billion (the approximate median number of bases in all segments for each sample across all analyzed samples and tumor types).

Cell-free DNA extraction

Five milliliters of peripheral blood were collected for 92 samples from 76 patients with metastatic castration resistant prostate cancer (mCRPC) and 10 healthy controls (5 male, 5 female) using K2 EDTA blood collection tubes (Cat: 366643, BD, NJ) (Table S1). Within 4 hr, blood was mixed with equal volume of PBS and Ficoll-Paque Plus (Sigma-Aldrich; MO) was used to separate plasma from red blood cells and peripheral mononuclear cells (PBMC). Plasma was centrifuged twice at 1500 g for 12 min to limit cell contamination and stored in −80°C.

For 11 patients (13 samples) with metastatic lung adenocarcinoma, 4 patients (7 samples) with metastatic colorectal cancer, 3 patients (3 samples) with leukemias, and 2 patients (4 samples) with sarcoma, one patient with both sarcoma and breast cancer, and a patient with uterine leiomyosarcoma, 10 mL peripheral blood was collected using Streck Cell-Free DNA BCT tube (Streck; NE) (Table S1). Within 4 hr, blood was centrifuged at 1600 g for 10 min, and then plasma was centrifuged at 1600 g for 10 min to remove cell debris and stored in −80°C. Cell free DNA was extracted from all plasma (2 mL) samples with QIAamp Circulating Nucleic Acid Kit (Qiagen; CA) according to the manufacturer's instructions. Sample collection and NGS was performed with Institutional Review Board approval.

Low-pass whole-genome sequencing and copy-number detection

Sequencing alignment and coverage analyses were performed using Torrent Suite version 5.0.2 (Ion Torrent, Carlsbad, CA). Initially, reads were aligned to the hg19 version of the human reference genome using tmap (v5.0.7) and aligned, non-PCR-duplicate reads (samtools v1.3) were used as input for our copy-number calling workflow. Genome-wide copy number alterations were first called using the QDNASeq R package (version 1.6.1) [2]. Briefly, the genome was divided up into variable bin sizes (15, 25, 50, 100, 500, and 1,000 kilobase-pair bins), and bin-level counts of high-quality mapped reads (MAPQ ≥ 37) were calculated separately for each sample. Raw bin-level counts were simultaneously corrected for GC content and mappability by fitting a LOESS surface through median read counts for bins with the same combination of GC content and mappability and dividing raw bin-level counts by the corresponding LOESS fitted value. GC- and mappability-corrected bin-level counts were then normalized by median bin-level corrected counts within each sample. Bins previously shown using either ENCODE or 1000G data to yield anomalous copy-number results due to germline copy number variants (CNVs), low mappability, or large stretches of uncharacterized nucleotides were excluded [2]. For each bin in each tumor sample, high-quality, corrected, median-normalized read counts were divided by average corrected, median-normalized read counts from our 5 normal male samples. Segmented copy-number events were called from bin-level corrected, median- and control-normalized read counts using the circular binary segmentation algorithm implemented by the DNACopy (1.44.0) R package, and final segment- and bin-level copy-number values were used for subsequent analyses as described. Focal CNAs were defined as CNAs 1.5–20 Mb long with a log2(CNRatio) ≥ 0.2, thresholds similar to those described elsewhere [3].

Targeted sequencing: oncomine comprehensive assay

For 60 patient cfDNA samples (31 high tumor content mCRPC samples, 13 low tumor content mCRPC samples, 11 high tumor content non-mCRPC samples, 1 mCRPC sample with germline chr20 deletion, and 4 male normals; see Table S1) and both sheared UMUC-5 and VCaP gDNA samples, we performed targeted NGS using the DNA component of the Oncomine Comprehensive Assay (OCP), a custom multiplexed PCR-based panel of 2,530 amplicons targeting 126 genes. These genes were selected based on pan-cancer analysis that prioritized somatic, recurrently altered oncogenes, tumors suppressors and genes subject to high level copy alterations, combined with a comprehensive analysis of known/investigational therapeutic targets [4]. Barcoded libraries were generated from 1–20 ng of cfDNA per sample and multiplexed sequencing was performed using the Ion Torrent Proton sequencer. Library preparation with barcode incorporation, template preparation on the OneTouch 2 and sequencing using the Ion Torrent Proton sequencer (Ion Torrent, Carlsbad, CA) were performed according to the manufacturer's instructions. Data analysis was performed using Torrent Suite 5.0.2, with alignment by TMAP using default parameters, and variant calling using the Torrent Variant Caller plugin (5.0.2.1) using default low-stringency somatic variant settings. Variant annotation filtering and prioritization, along with gene-level copy number estimation, were performed essentially as described [4–7] using validated in house pipelines, and gene level copy-number calls, and prioritized point mutations, small insertions/deletions (indels), and copy-number variants were reported for each patient sample (Table S2 & S3). Copy number alterations called from targeted NGS data with log2(copy number ratio) >= 0.6 or <= -1.0 were prioritized.

VCaP and UMUC-5 *In silico* dilution

To establish theoretical segment-level copy-number distributions for tumor content estimation, we carried out serial *in silico* dilution experiment by mixing read proportions derived from undiluted VCaP and UMUC-5 whole-genome sequencing data and our set of normal male patient samples. Briefly, we combined FASTQ files for the whole-genome sequencing experiments from our 5 normal male samples (n=85,981,532 total unaligned reads). We then shuffled reads (*“awk ‘{OFS=\”\\t\”; getline seq; getline sep; getline qual; print \$\$0,seq,sep,qual}’ <norm_fastq_file> | shuf | awk ‘{OFS=\”\\n\”; print \$\$1,\$\$2,\$\$3,\$\$4}’*), and randomly sampled an identical number of non-PCR-duplicated reads as was present for the VCaP (n=6,670,015 reads; whole-genome coverage= 0.26x) and UMUC-5 (n=16,570,486 reads; whole-genome coverage = 0.74x) undiluted whole-genome sequencing samples.

In silico dilutions were subsequently carried out on both undiluted whole-genome sequencing cell line samples with our coverage-matched normal male sample (for all integer percent dilutions 0–100%), where for each dilution the following steps were executed:

1) Shuffle undiluted cell line & normal male FASTQ files (using code above)

2) Sample appropriate portion of reads from each file using seqtk NGS toolkit (v1.0-r31) (*seqtk sample –s100 <FASTQ file><proportion_to_sample>*)

3) Concatenate proportional FASTQ files (*cat <vcap_prop_file><normal_prop_file>*)

4) Map mixed read set to the reference genome (hg19) using identical mapping approach to that used for original undiluted cell line and patient whole-genome sequencing samples:

tmap mapall –f hg19.fasta –r input.fastq –s output.bam –v –Y –u –prefix-exclude 5 –o 1 stage1 map 4

5) Sort and index aligned bam files for input to copy-number calling workflow

Genome-wide copy number variation calls were subsequently generated for each *in silico* dilution as described (see Methods).

Clustering

Mean-shift, k-means, and xmeans clustering approaches were assessed and deployed to identify relevant clusters from segment- (whole-genome sequencing) or gene-level (targeted sequencing) copy number ratio data. All clustering analyses were carried out in R (3.2.3) using packages LPCM (v0.45–0), RWeka (0.4–26), or base packages as applicable. For mean-shift clustering, variable bandwidths were evaluated, supporting a static bandwidth value of 0.01 on exome or whole-genome copy-number calls. Mean-shift clustering showed the most consistent expected cluster identification across *in vitro*/*in silico* dilutions, and was used for all analyses described herein.

Tumor content estimation

For whole-genome sequencing samples, reference segment-level copy-number ratio distributions were established through serial *in vitro* and *in silico* VCaP and bladder (UMUC-5) cell line dilutions as described. A heuristic least squares based distance metric (LSS) was used to approximate tumor content from whole-genome copy-number data. LSS between cluster centroids was calculated as a proxy for tumor content using the following formula:

$$\sum_{i=2}^{n} \sqrt{a[i]^\wedge 2 - a[i-1]^\wedge 2}$$

where is the vector of cluster centroids for clusters identified by the mean-shift algorithm, is the length

of the cluster vector, and is th element of this vector. If only one cluster was assigned for a given sample, LSS was calculated as the square root of the cluster center squared (equivalently, the absolute value of the cluster centroid):

$$\sqrt{cluster\_center^{\wedge}2} = |\text{cluster\_center}|$$

Reference LSS distributions were established across serial *in silico* dilution experiments at all integer percent dilutions 0–100% as described, and these distributions were used to guide tumor content estimation for patient samples. While tumor content estimates were not generated for samples with LSS values < 0.1, these samples were specifically scanned for focal CNAs, as described above.

In silico experiments: downsampling

For the VCaP and UMUC-5 *in silico* dilutions, as well as 9 patient cfDNA samples (5 w/highest tumor content, 1 germline chr21 deletion, 2 no tumor content), we carried out *in silico* downsampling experiments to evaluate capacity to call copy-number alterations at variable effective whole-genome coverages (range: 0.005–0.1×). After downsampling (using *samtools view –s <proportion of reads to sample> –bh <original.bam.file>*) for each sample, copy-number alterations were called across variable bin sizes as described. Given the effective coverages analyzed, bin sizes were not analyzable across all coverages (e.g., 0.01× whole-genome coverage corresponds to approximately 150 k single-end reads, leaving < 10 reads per 100 kb bin, on average). For this reason, we considered effective coverage & bin combinations ≥ 30 reads per bin as analyzable for this analysis.

Serial *in silico* downsampling experiments were also carried out on targeted sequencing data from 10 mCRPC patient plasma cfDNA samples (5 high tumor content, 1 germline chr20 deletion, and 3 normals) to 500, 250, 100, 50, and 25x effective target coverage by the same sampling approach taken with whole-genome data.

VCaP cfDNA WGS vs COSMIC array-based CN calls

Of 500 segment-level copy-number calls for chromosomes 1–22 & X reported as present in VCaP by COSMIC, 464 (92.8%) overlapped ≥ 90% of at least one 15kbp bin from our low pass (0.26x whole-genome coverage) analysis of undiluted VCaP, with 496 (99.2%) showing at least some (≥ 1 bp) overlap of one bin or more. We calculated median of bin-level integer copy number values for all 15kbp bins overlapped at ≥ 90% by a COSMIC-reported copy-number segment, and compared these low-pass sequencing derived values to segment-level integer copy-number values reported in COSMIC. Given the known variability in reported copy-number estimates for VCaP focal AR amplification (copy number of 14 reported by COSMIC; at least 3–18 copies by FISH [8]), we explored correlations between COSMIC segmented copy-number and both raw and capped (copy-number = 14) sequencing-derived copy-number values.

UMUC-5 cfDNA WGS vs Targeted NGS CN Calls

Copy number calls from whole genome sequencing of sheared UMUC-5 genomic DNA (gDNA) were compared to calls derived from targeted sequencing (OCP) of sheared DNA in this study. Of 126 genes targeted on the OCP, 90 had more than 3 amplicons and amplicon-level estimate variability sufficient for gene-level copy-number analysis. Coding sequence for 87/90 genes (97%) overlapped at least one 15 kbp bin-level call from whole-genome sequencing data of sheared gDNA. Gene-level copy number estimates from whole-genome sequencing data were calculated as mean log2 copy number ratio for 15 kb bins overlapping genome space from first to last coding base pair for each of the 87 genes.

Application to exome sequencing segmented copy-number calls

In order to test the efficacy of this particular approach for approximating tumor content from alternate datasets, we tested our LSS approach on segmented-copy-number calls from 129 clinical advanced/treatment refractory cancer tissue samples subjected to exome sequencing as part of the MI-ONCOSEQ project at the University of Michigan [9, 10]. Tumor content for all MI-ONCOSEQ samples is estimated through a model fitting variant allele frequencies of all somatic mutations and a model assessing zygosity shift of heterozygous SNPs and local copy number [9, 10]. As our analysis of TCGA copy-number data, the fraction of genome altered (FGA) was calculated for each MI-ONCOSEQ sample as the total number of bases in regions affected by segmented copy-number alterations with log2(CopyNumberRatio) > 0.2 or < -0.2 divided by 3 billion (the approximate median number of analyzable bases across all analyzed samples).

Concordance with tissue-based whole-exome sequencing copy-number profiles

Segmented log2 copy number ratio data from whole-exome sequencing of fresh frozen tissue specimens [9, 11] was available for 23 of 27 patients also profiled by cfDNA low-pass WGS. Each of these 23 patients had at least 1 cfDNA plasma sample (range: 1–3), and 18 of 23 (78.3%) had at least 1 cfDNA sample with elevated tumor content (LSS ≥ 0.1) suitable for concordance analyses. For these 18, the median of cfDNA low-pass WGS bin-level copy number values for all 500kbp bins overlapped at ≥ 90% by a tissue-based copy-number segment was calculated as

a pseudo-cfDNA segment call, and correlations between tissue- and cfDNA-based copy number ratios were evaluated.

Focal AR amplification determination

Given the difficulty of appropriate copy number segmentation on chrX, median 100kb bin-level copy number estimates across chrX q-arm were subtracted from mean 100kb bin-level copy-number estimates at *AR* locus (chrX:66.0–67.5Mb), and difference values >= 0.2 were used to call focal *AR* amplifications in our mCRPC cohort. Two cfDNA high tumor content samples (TP1216 and TP1295) met the above criteria, but were excluded as potential false positives due to use of 100kb bin width at low coverage (< 300,000 total high-quality (MAPQ >= 37) mapped reads). An additional low tumor content sample (TP1139) met the amplification criteria, but with excessive variability in chrX bin-level copy-number estimates, was considered negative for *AR* amplification for all subsequent analyses.

RESULTS

Validation of ThruPLEX cfDNA WGS for Ion Torrent Benchtop Sequencers

Given the limited amount of recoverable cfDNA requiring amplification for WGS, we first sought to validate the performance of ThruPLEX RGP-0003 WGA library construction with index barcode and adaptor incorporation for rapid sequencing on Ion Torrent benchtop sequencers. This single tube approach is compatible with ≤ 50pg double stranded DNA and generates libraries with Ion Torrent barcode index and sequencing adaptors in a three hour workflow. Using the Ion Torrent Proton sequencer, we sequenced pooled ThruPLEX cfDNA libraries from normal controls ($n = 10$), resulting in an average genome wide coverage depth of 0.62× (range: 0.16–1.30×), with no evidence of differential coverage in males (mean: 0.63× [0.17–1.30×]) vs. females (mean 0.61× [0.16–1.03×], two-sided t-test: t = 0.046, $p = 0.965$, Supplementary Table 1). All ThruPLEX cfDNA libraries from normal controls showed high sequencing coverage uniformity (mean uniformity: 93.9%; range: 90.8–97.0%). ThruPLEX cfDNA libraries from advanced cancer patient samples ($n = 123$) were sequenced to an average depth of 0.31x genome-wide (0.01–1.30×), at comparable uniformity (96.2%, (range: 90.8–99.7%)) to normal controls.

We next sought to validate the ability of our low pass WGA cfDNA profiling approach to robustly detect genome-wide copy-number profiles to facilitate direct management (if CNAs are actionable) and guidance of additional testing through cfDNA tumor content approximation. Using our WGA cfDNA profiling approach, *in vitro* dilutions of sheared genomic DNA for VCaP and UMUC-5 cell lines were sequenced to an average genome wide coverage depth of 0.72x (range: 0.19–1.15x) with average sequencing uniformity of 91.6% (range: 90.8–96.8%) (Supplementary Table 1). As shown in Supplementary Figure 2A, the genome-wide copy-number profile from low-pass WGS of an undiluted artificial VCaP cfDNA sample (genomic DNA sheared to ~180bp) revealed known broad and focal CNAs, including characteristic 8p loss/8q gain, focal *AR* amplification, and evidence of the previously-reported chromothripsis event on 5q [12]. We then compared regional overlap and magnitude of our low-pass WGS segmented copy number calls from the undiluted artificial VCaP cfDNA sample to those reported in COSMIC for VCaP [13]. We observed a highly significant correlation between our median segment-level low-pass sequencing-derived copy number calls from the undiluted VCaP sample and copy-number values reported in COSMIC (Pearson correlation = 0.77, $p < 0.001$), with even stronger correlation when the cfDNA copy-number estimation of the highly amplified *AR* locus was capped (see Supplementary Methods; Pearson correlation = 0.92, $p < 0.001$) (Supplementary Figure 2B). Likewise, we observed significant correlation between gene-level copy number estimates from a validated targeted multiplexed PCR based approach [4] on genomic DNA from the UMUC-5 urothelial carcinoma cell line compared to artificial UMUC-5 cfDNA subjected to ThruPLEX library preparation (Pearson correlation coefficient = 0.92, $p < 0.001$; see Supplementary Figure 2). Together, these results support the fidelity of copy number profiling from cfDNA using ThruPLEX library preparation and low coverage Ion Torrent sequencing.

Assessment of copy number calling robustness from ultra low pass cfDNA WGS across varying tumor content

Accurate approximation of tumor content from cfDNA is critical to using low-pass WGS to guide management based on low-pass WGS profiles. Hence, we next used our in vitro and in silico dilution series to develop a novel tumor content approximation approach (LSS) that can robustly approximate tumor content in cfDNA samples with tumor content > ~10% (Figure 2C). Our approach leverages the distribution of segment-level copy number ratios to inform on cfDNA tumor content; specifically, we use the expectation that as tumor content decreases, so too should the distance between peaks (or 'clusters') in segment-level copy-number ratio distributions (Supplementary Figure 3A and 3B). After clustering of segment-level copy-number ratios and identification of cluster centers, our LSS metric aggregates the distance between cluster centers to infer cfDNA tumor content from low-pass WGS genome-wide copy number calls using known *in vitro* and *in silico* dilutions (see Supplementary Figure 4 and Supplementary Methods). Supplementary Figure 3C highlights the near-linear

relationship between LSS and effective tumor content across both VCaP and UMUC-5 *in silico* dilutions, enabling application of these reference LSS distributions for interpretation of LSS values and approximation of tumor content from WGS cfDNA patient samples described below. In addition, as shown in Supplementary Figure 3D, we also found that our clustering and LSS approach applied to segmented genome wide copy-number profiles from the 129 MI-ONCOSEQ profiled advanced cancer tissue samples (see Fig 1B) was highly concordant to tumor content estimates made in MI-ONCOSEQ using exome-wide SNVs and heterozygous SNPs. Taken together, these results confirm our ability to detect clinically relevant focal CNAs (such as *EGFR* and *AR* amplifications) down to 5% effective cfDNA tumor content, determine genome-wide copy number profiles of both focal and broad CNAs, and approximate tumor content from ThruPLEX cfDNA libraries.

We next sought to validate our ability to detect genome-wide CNAs across a range of effective cfDNA tumor content for ultra low pass WGS (0.005–0.1x whole-genome coverage) using *in vitro* dilution data and further *in silico* dilution experiments (see Supplementary Methods). Across both the UMUC-5 and VCaP *in vitro* dilution series, genome-wide bin- and segment-level copy-number ratio estimates were generated and both focal and broad copy-number alterations were detected (Supplementary Figures 2, 5, and 7). For VCaP, significant correlations ($p < 0.05$) were observed for segment-level copy-number values down to effective tumor contents of 10% and 5% for *in vitro* and *in silico* dilutions respectively, suggesting our approach is capable of systematically detecting genome-wide copy number profiles even at low tumor content (see Supplementary Figure 2). As high level focal amplifications are the majority of actionable CNAs, we also focused on the known focal *AR* amplification in VCaP, along with the known focal *EGFR* amplification in UMUC-5 [14, 15], which could both be robustly detected down to 5% tumor content based on *in vitro* and *in silico* dilutions (Supplementary Figure 7). For UMUC-5, we observed a similar ability to identify both focal and broad copy number alterations at expected copy-number ratios across the full *in vitro* dilution series (Supplementary Figure 5). Significant correlations between bin-level sequencing-derived copy number calls and gene-level copy number calls derived from targeted NGS for the UMUC-5 cell line were also observed across the full *in vitro* dilution series ($p < 0.001$; see Supplementary Figure 5).

PRINCe concordance with comprehensive tissue-based profiling

To systematically evaluate concordance for somatic molecular alterations across multiple biocompartments, we focused on 27 of the 76 men (35.5%) with mCRPC already profiled by cfDNA low-pass WGS (corresponding to 33 of 93 (35.5%) mCRPC cfDNA samples) where synchronous or asynchronous comprehensive whole exome and whole transcriptome profiling was attempted on fresh frozen or FFPE tissue specimens. Of 27 men, 4 (14.8%) had either insufficient tumor content for comprehensive profiling by biopsy or incomplete tissue profiling data for analysis. Notably, all 4 men had cfDNA samples that yielded clinically informative results. TP1182 [TP_2007] was a plasma cfDNA sample taken 2 years after MiOncoseq biopsy (MiOncoseq biopsy reported 10% tumor content by pathology review, and no DNA sequencing was done on MiOnco FFPE tissue), and by plasma cfDNA profiling demonstrated a focal AR amplification and focal 2-copy *PTEN* deletion. TP1201 and TP1405 [TP_2278] were plasma cfDNA samples taken 2 years and 1 month prior to Mi-Oncoseq research biopsy of metastatic bone lesion, respectively. Tissue biopsy yielded < 10% tumor content by pathologist review and was not profiled by DNA or RNA sequencing. While TP1201 (taken two years prior to tissue biopsy) had no detectable cfDNA tumor content nor focal copy-number alterations, TP1405 (taken 1 month prior to the research biopsy) shows low (but detectable) tumor content and detectable focal *AR* amplification.

Another plasma cfDNA sample (TP1353 [MO_1579]) was taken 7 months after a Mi-Oncoseq tissue biopsy that yield 6 tissue blocks w/no tumor content, while low-pass cfDNA WGS revealed a focal *AR* amplification and 2-copy *PTEN* deletion. Both copy-number alterations were validated by targeted NGS of the same cfDNA sample, and *TP53* G245S (variant fraction: 32.7%) and *ATM* D817H (28%) mutations were also detected. Lastly, TP1354 [TP_2171] was taken 1 week before a Mi-Oncoseq bone needle core biopsy that yielded no tumor by pathology review (‘blind sequencing’ of DNA & RNA was still attempted). While no somatic alterations were identified by comprehensive tissue-based profiling of the bone needle core biopsy sample, cfDNA low-pass WGS and targeted NGS identified both a focal *AR* amplification, as well as *TP53* R282W (14.2%) and *KDR* T1336I (5.4%) somatic mutations. These results highlight potential complementary clinical utility for plasma cfDNA profiling in comprehensive tissue-based NGS workflows.

Of 23 men with comprehensive tissue-based profiling and at least 1 profiled cfDNA sample (range of cfDNA samples per individual: 1–3), 20 (87%) had a cfDNA sample w/elevated cfDNA tumor content amenable to analysis. To evaluate concordance between cfDNA- and tissue-based DNA copy-number profiles, segmented tissue-based copy-number profiles were compared to whole genome cfDNA segmented copy-number profiles for the 18 of 20 (90%) individuals with fresh frozen tissue specimens within 2 years of blood sample collection (see Supplementary Methods). Low-pass whole-genome WGS copy-number profiles were highly correlated (median r = 0.86 [range: 0.54–0.94) for these samples despite variable specimen tumor content and sample collection synchronicity (median number days between tissue- and cfDNA specimen collection: 108 (range: 0–682 days)). One patient (TP_2073/TP1303) with synchronous tissue and blood specimens displayed both high correlation of

genome-wide copy-number profiles (Pearson corr: 0.96), as well as fully concordant somatic mutations for regions targeted by both tissue whole exome and cfDNA targeted NGS approaches, including a putative homozygous *TP53* splice mutation (p.R2494X) present at 89% in tissue and 49% in cfDNA (VF: 48.5%, 414/853 flow-corrected variant-containing reads). Further, 5 of 10 patient samples with clear 21q22.2 copy-number deletion consistent with deletion leading to TMPRSS2:ERG gene fusion were from patients with tissue-based whole transcriptome profiling, and *TMPRSS2:ERG* fusion isoform expression was confirmed in 5/5 (100%) corresponding tissue specimens.

In total, 17/58 (29.3%) high tumor content cfDNA samples demonstrate detectable focal *PTEN* deletions, of which 11 (64.7%) were likely 2-copy losses. Of these, 4 had near-synchronous analyzable tissue-based profiling data, and all 4 corresponding tissue-based copy-number profiles show focal deep / likely 2-copy *PTEN* deletions. Copy number losses affecting *RB1* were also frequent in our high tumor content cohort, with 4 samples (4 patients) exhibiting focal 2-copy *RB1* deletions. While 3 of these 4 patients were lost to follow-up, the remaining patient (TP1320) also had detectable *AR* amplification, and having received a single (taxel-based) line of therapy post-ADT, progressed rapidly on abiraterone over the course of 3 months on therapy (PSA rising from 37.3 to 70.4), with PCa-related death 4 months after cfDNA profiling (Supplementary Table 1). Another patient (TP1282) also underwent comprehensive synchronous tissue profiling (MO_1473) of a left femur bone biopsy 2 weeks after blood collection, and while demonstrating high overall copy-number concordance (Pearson corr: 0.94), tissue profiling identified only a 1-copy *RB1* loss compared to the 2-copy loss seen by cfDNA low-pass WGS. Overall, given the known frequency of *RB1* hemi- and homozygous copy number loss in advanced and castration-resistant neuroendocrine/small-cell prostate cancer [11, 16], these results highlight our capacity to detect therapeutically relevant focal copy-number deletions from low-pass WGS of cfDNA from routine blood samples.

While high levels of overall genome-wide concordance were observed between tissue- and plasma cfDNA-based copy-number profiles, discrepancies with important clinical relevance were also identified. In one patient with a history of both primary prostatic adenocarcinoma and a metastatic lesion with small cell carcinoma/neuroendocrine features (TP1019/MO_1234), synchronous profiling of same-day specimens detected a clear focal *AR* amplification in the cfDNA that was absent in the tissue based profiling of a small cell carcinoma focus (despite identical prioritized somatic point mutations), suggesting circulating evidence of both *AR*-driven and *AR*-independent clones. Further, applications of this approach in advanced, treatment-naïve patients have also suggested utility for identifying clinically relevant copy-number changes (including focal 2-copy *PTEN* and *RB1* deletions) in patients with high tumor burden. Overall, these results suggest noninvasive profiling may yield high concordance with near-synchronous tissue profiling for clinically relevant molecular alterations, and can provide unique complementary advantages and opportunities for expansion into treatment-naïve patient cohorts.

PRINCe in other cancers and for disease monitoring

To demonstrate feasibility and potential applicability of PRINCe for actionable CNA detection and disease monitoring in other tumor types, we also assessed 31 plasma cfDNA samples from 24 patients with other cancers (including lung, breast, colon, and sarcomas) (Supplementary Table 1). Of these, 8 samples from 6 unique patients had known mutations as determined by previous ddPCR (Supplementary Table 1). Importantly, PRINCe estimated cfDNA tumor content was highly concordant with ddPCR findings, with all 4 solid tumor cfDNA samples harboring ddPCR detected mutations at >4% variant fraction also being estimated by PRINCe as having high tumor content (> 8.75%, LSS > 0.1). For example, PRINCe analysis of cfDNA from ULMC-128 (a patient with lung adenocarcinoma) identified a focal *EGFR* amplification, despite relatively low estimated cfDNA tumor content (~10%). Previous analysis by ddPCR identified an *EGFR* exon 19 deletion (c.2236_2250del15, p.E746_A750del) at 36.2% variant fraction, consistent with amplification of mutant *EGFR*. This deletion was also seen by cfDNA targeted NGS (variant fraction = 59.0%, FAO/FDP = 262/444).

Our non-mCRPC cohort also contained paired pre- and post-treatment cfDNA samples from several patients. For example, we profiled pre- and post-EGFR inhibitor treatment initiation cfDNA samples from ULMC-125 (a patient with metastatic lung cancer). By PRINCe, ULMC-125's pre-treatment sample showed focal *EGFR* and *FGFR1* amplifications and multiple arm-level and whole chromosome gains and losses, while previous ddPCR on the cfDNA identified an activating *EGFR* L858R hotspot mutation at a 62.5% variant fraction (consistent with amplification of mutant *EGFR*) (Figure 5E). PRINCe analysis of ULMC-125's post-treatment cfDNA sample showed no detectable CNAs, consistent with no detectable *EGFR* L858R by ddPCR. PRINCe analysis of serial pre- and post-treatment cfDNA samples for ULMC-151 and ULMC-194 (patients with colorectal adenocarcinoma) also showed substantial depletion in detectable genome-wide CNAs in the post-treatment samples, consistent with reduced (though non-absent) tumor-derived cfDNA fragment representation.

Low-pass WGS and targeted NGS profiling of a pre-treatment plasma sample (PD-L1006_1) from a patient with stage IV lung adenocarcinoma who subsequently achieved a complete response after two doses of a PD-L1 inhibitor revealed high-level focal amplifications of *CCND3* and *CD93*, along with a *KRAS* G12C hotspot mutation (6.4%, 29/456 variant containing reads). Three consecutive weekly plasma samples taken 5 months after

completion of palliative radiotherapy from ULMC-185, an individual with a history of neurofibromatosis type 1 and pelvic sarcoma, highlight consistently elevated cfDNA tumor content and detectable focal *EGFR* amplification in each plasma sample, and putative germline *ATM1* (I124V) and *BAP1* (T423K) mutations in each sample. Together these results suggest substantial potential clinical utility for treatment response and disease monitoring using highly scalable complementary whole-genome and targeted cfDNA NGS-based profiling strategies.

These results further reinforce our ability to detect therapeutically relevant alterations across multiple cancer types using low-pass WGS of patient plasma cfDNA, even at tumor contents as low as 10%. Likewise, although our approach will obviously not be able to detect molecular evidence of disease recurrence at ultra-low tumor content (in contrast to ultra-deep, extremely accurate or personalized sequencing/ddPCR methodologies [17–19]), it can identify pre-treatment genome wide CNA profiles and cfDNA tumor content estimates that may enable low-cost and more frequent assessment of molecular evidence of recurrence in post-treatment cfDNA samples.

PRINCe as a precision medicine screening strategy

Given the above results demonstrating the utility of our approach for cfDNA based CNA profiling and tumor content approximation with very low coverage (~0.1–1×), we next sought to determine the robustness of ultra-low pass WGS (0.005–0.1x coverage) in order to decrease sequencing costs per sample. Down-sampling across our cell line and mCRPC samples demonstrated that our approach robustly determined high-quality whole-genome copy-number profiles down to 0.005x whole-genome coverage. For example, Supplementary Figure 6 depicts the CNA profile across effective whole genome coverages down to 0.005× (~82,000 reads) for patient sample TP1337, with robust detection of both broad and focal clinically relevant CNAs. Overall, we show this method performs well at 0.005x coverage down to effective tumor contents of ~10%, though accurate approximation of tumor content (vs. detection of CNAs) is challenging at such low coverage/tumor content. Importantly, however, we observed that the high level, focal *EGFR* amplification in UMUC-5 cells, as well as the *AR* amplification observed in VCaP cells and 8 of 9 (89%) high tumor content mCRPC samples, can be robustly detected at 0.01x coverage. At 0.005× coverage, although automated detection of *AR* amplification is less reliable, bin level copy number estimates demonstrate clear amplifications in the majority of samples. Taken together, our results support our ultra-low pass cfDNA WGS based PRINCe approach as capable of estimating tumor content from genome wide copy number profiles as well as identifying high level focal amplifications, a key therapeutic class of somatic alterations in cancer.

PRINCe to guide additional precision oncology testing

Although ultra-low pass cfDNA WGS is capable of detecting high level CNAs at relatively low tumor contents, additional approaches are needed to detect other alteration classes (mutations, short insertion/deletions and chromosomal rearrangements) and in patients with very low tumor content. As shown in Figure 1C, PRINCe approximation of cfDNA tumor content can be used to guide additional precision medicine in patients without potentially actionable/informative CNAs. For example, in patients with high tumor content by ultra-low pass cfDNA WGS, additional routine targeted sequencing, exome sequencing or WGS could all be performed on the cfDNA (or WGA cfDNA library), with coverage informed by the estimated tumor content, while ultra-deep cfDNA sequencing (or sequencing a tissue sample) can be reserved for patients with very low cfDNA tumor content.

To demonstrate the potential utility and feasibility of PRINCe in guiding such additional testing, we subjected separate 1–20 ng aliquots of unamplified cfDNA from 61 of our patient samples (32 high tumor-content and 14 low tumor-content mCRPC samples, 11 high tumor content non-mCRPC samples, and 4 male control samples with sufficient DNA), as well as the undiluted artificial VCaP and UMUC5 cfDNA samples as positive controls, to targeted multiplexed PCR based NGS using the DNA component of the Oncomine Cancer Assay (OCP). The OCP assay targets 126 potentially actionable tumor suppressors and oncogenes recurrently altered across cancers; we have previously validated this assay for somatic mutation and copy number calling from FFPE isolated DNA [4].

Sequencing of pooled patient samples resulted in a median average coverage of 1,075x (range: 42–17,944x), with average uniformity of 96.0% (higher than typically observed for FFPE DNA samples [4]). OCP on cfDNA confirmed high level *EGFR* amplification in UMUC-5, and high level *AR* amplifications in VCaP and all 22 high tumor content mCRPC samples sequenced (Supplementary Table 3). OCP sequencing of TP1337 cfDNA validated high-level *AR* amplification, focal two-copy *PTEN* deletion, 1 copy *RB1* deletion, and 8q gain originally identified by low-pass cfDNA WGS, and enabled detection of a unique somatic 28bp *TP53* frameshift deletion (L264del28bp, variant allele frequency 20.8% with 504 covering reads) (Supplementary Figure 6). Critically, we observed high correlation between gene-level copy number alterations by targeted sequencing and segment-level calls in patient cfDNA samples from PRINCe (Pearson correlation coefficient: 0.80, $p < 0.001$). OCP sequencing of TP1291 cfDNA validated 1-copy *PTEN* and *BRCA2* copy-number loss, and 2-copy *RB1* deletion, as well as focal *AR* amplification observed in cfDNA low-pass WGS. Interestingly, targeted NGS of TP1291 cfDNA also detected a known Clinvar pathogenic stop-gain SNV at

71.1% variant fraction (p.R2494X, 1022/1437 variant containing reads), consistent with copy-number loss of the wild-type *BRCA2* allele. Further, we identified the known homozygous *TP53* R248W missense SNV in the VCaP sample (observed variant allele frequency 95.3%; 657× flow corrected coverage), as well as somatic, prioritized *TP53* mutations in all 5 (100%) high tumor content mCPRC patient samples sequenced (see Supplementary Table 4). *In silico* down-sampling experiments in targeted NGS data suggest mean coverages as low as 50× enable reliable detection of known putative clonal somatic point mutations, indels, and copy number variants in UMUC-5 simulated cfDNA and patient cfDNA samples with high tumor content (Supplementary Figures 12 and 13). Taken together, these results underscore the potential for PRINCe followed by targeted sequencing (tuned to cfDNA tumor content) as part of a high-throughput, cost-effective clinical or translational research NGS workflow.

REFERENCES

1. Analysis-ready standardized TCGA data from Broad GDAC Firehose 2016_01_28 run., in Center BITGDA (ed). Broad Institute of MIT and Harvard. 2016.
2. Scheinin I, Sie D, Bengtsson H, van de Wiel MA, Olshen AB, van Thuijl HF, van Essen HF, Eijk PP, Rustenburg F, Meijer GA, Reijneveld JC, Wesseling P, Pinkel D, et al. DNA copy number analysis of fresh and formalin-fixed specimens by shallow whole-genome sequencing with identification and exclusion of problematic regions in the genome assembly. Genome Res. 2014; 24:2022–32.
3. Ulz P, Belic J, Graf R, Auer M, Lafer I, Fischereder K, Webersinke G, Pummer K, Augustin H, Pichler M, Hoefler G, Bauernhofer T, Geigl JB, et al. Whole-genome plasma sequencing reveals focal amplifications as a driving force in metastatic prostate cancer. Nat Commun. 2016; 7:12008.
4. Hovelson DH, McDaniel AS, Cani AK, Johnson B, Rhodes K, Williams PD, Bandla S, Bien G, Choppa P, Hyland F, Gottimukkala R, Liu G, Manivannan M, et al. Development and validation of a scalable next-generation sequencing system for assessing relevant somatic variants in solid tumors. Neoplasia. 2015; 17:385–99.
5. Cani AK, Hovelson DH, McDaniel AS, Sadis S, Haller MJ, Yadati V, Amin AM, Bratley J, Bandla S, Williams PD, Rhodes K, Liu CJ, Quist MJ, et al. Next-Gen Sequencing Exposes Frequent MED12 Mutations and Actionable Therapeutic Targets in Phyllodes Tumors. Mol Cancer Res. 2015; 13:613–9.
6. McDaniel AS, Hovelson DH, Cani AK, Liu CJ, Zhai Y, Zhang Y, Weizer AZ, Mehra R, Feng FY, Alva AS, Morgan TM, Montgomery JS, Siddiqui J, et al. Genomic Profiling of Penile Squamous Cell Carcinoma Reveals New Opportunities for Targeted Therapy. Cancer Res. 2015; 75:5219–27.
7. McDaniel AS, Stall JN, Hovelson DH, Cani AK, Liu CJ, Tomlins SA, Cho KR. Next-Generation Sequencing of Tubal Intraepithelial Carcinomas. JAMA Oncol. 2015; 1:1128–32.
8. Liu W, Xie CC, Zhu Y, Li T, Sun J, Cheng Y, Ewing CM, Dalrymple S, Turner AR, Sun J, Isaacs JT, Chang BL, Zheng SL, et al. Homozygous deletions and recurrent amplifications implicate new genes involved in prostate cancer. Neoplasia. 2008; 10:897–907.
9. Roychowdhury S, Iyer MK, Robinson DR, Lonigro RJ, Wu YM, Cao X, Kalyana-Sundaram S, Sam L, Balbin OA, Quist MJ, Barrette T, Everett J, Siddiqui J, et al. Personalized oncology through integrative high-throughput sequencing: a pilot study. Sci Transl Med. 2011; 3:111ra121.
10. Robinson DR, Wu YM, Vats P, Su F, Lonigro RJ, Cao X, Kalyana-Sundaram S, Wang R, Ning Y, Hodges L, Gursky A, Siddiqui J, Tomlins SA, et al. Activating ESR1 mutations in hormone-resistant metastatic breast cancer. Nat Genet. 2013; 45:1446–51.
11. Robinson D, Van Allen EM, Wu YM, Schultz N, Lonigro RJ, Mosquera JM, Montgomery B, Taplin ME, Pritchard CC, Attard G, Beltran H, Abida W, Bradley RK, et al. Integrative clinical genomics of advanced prostate cancer. Cell. 2015; 161:1215–28.
12. Teles Alves I, Hiltemann S, Hartjes T, van der Spek P, Stubbs A, Trapman J, Jenster G. Gene fusions by chromothripsis of chromosome 5q in the VCaP prostate cancer cell line. Hum Genet. 2013; 132:709–13.
13. Forbes SA, Beare D, Gunasekaran P, Leung K, Bindal N, Boutselakis H, Ding M, Bamford S, Cole C, Ward S, Kok CY, Jia M, De T, et al. COSMIC: exploring the world's knowledge of somatic mutations in human cancer. Nucleic Acids Res. 2015; 43:D805–11.
14. Rebouissou S, Bernard-Pierrot I, de Reyniès A, Lepage ML, Krucker C, Chapeaublanc E, Hérault A, Kamoun A, Caillault A, Letouzé E, Elarouci N, Neuzillet Y, Denoux Y, et al. EGFR as a potential therapeutic target for a subset of muscle-invasive bladder cancers presenting a basal-like phenotype. Sci Transl Med. 2014; 6:244ra91.
15. Earl J, Rico D, Carrillo-de-Santa-Pau E, Rodríguez-Santiago B, Méndez-Pertuz M, Auer H, Gómez G, Grossman HB, Pisano DG, Schulz WA, Pérez-Jurado LA, Carrato A, Theodorescu D, et al. The UBC-40 Urothelial Bladder Cancer cell line index: a genomic resource for functional studies. BMC Genomics. 2015; 16:403.
16. Beltran H, Prandi D, Mosquera JM, Benelli M, Puca L, Cyrta J, Marotz C, Giannopoulou E, Chakravarthi BV, Varambally S, Tomlins SA, Nanus DM, Tagawa ST, et al. Divergent clonal evolution of castration-resistant neuroendocrine prostate cancer. Nat Med. 2016; 22:298–305.
17. Tie J, Wang Y, Tomasetti C, Li L, Springer S, Kinde I, Silliman N, Tacey M, Wong HL, Christie M, Kosmider S, Skinner I, Wong R, et al. Circulating tumor DNA analysis detects minimal residual disease and predicts recurrence in patients with stage II colon cancer. Sci Transl Med. 2016; 8:346ra92.
18. Newman AM, Lovejoy AF, Klass DM, Kurtz DM, Chabon JJ, Scherer F, Stehr H, Liu CL, Bratman SV, Say C, Zhou L, Carter JN, West RB, et al. Integrated digital error

suppression for improved detection of circulating tumor DNA. Nat Biotechnol. 2016; 34:547–55.

19. Garcia-Murillas I, Schiavon G, Weigelt B, Ng C, Hrebien S, Cutts RJ, Cheang M, Osin P, Nerurkar A, Kozarewa I, Garrido JA, Dowsett M, Reis-Filho JS, et al. Mutation tracking in circulating tumor DNA predicts relapse in early breast cancer. Sci Transl Med. 2015; 7:302ra133.

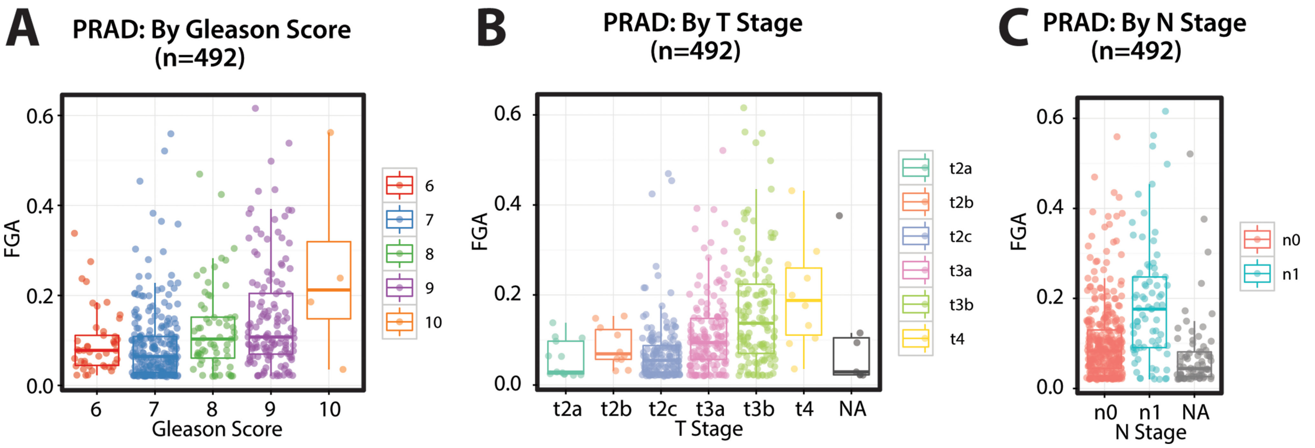

Supplementary Figure 1: Fraction of genome altered (FGA) analysis by stage/grade in TCGA prostate adenocarcinoma (PRAD) samples. Fraction of genome altered (FGA) analysis was carried out on 492 PRAD samples using segmented Affymetrix SNP6 array-based segmented calls extracted from the most recent standard analysis set generated by GDAC Firehose (stddata__2016_01_28). FGA was calculated for all PRAD tumor samples as the total number of bases in regions affected by copy-number alterations with log (base 2) copy number ratio (Log2CN) > 0.2 or < −0.2 divided by 3 billion (the approximate median number of bases in all segments for each sample across all analyzed TCGA samples and tumor types). (**A**) PRAD cohort FGA proportions are stratified by Gleason score, showing an increase in FGA as Gleason score increases. (**B** and **C**) PRAD cohort FGA proportions are stratified by tumor stage (B; T Stage) and clinical stage (C; N Stage), showing increased FGA in high stage and N stage disease as well.

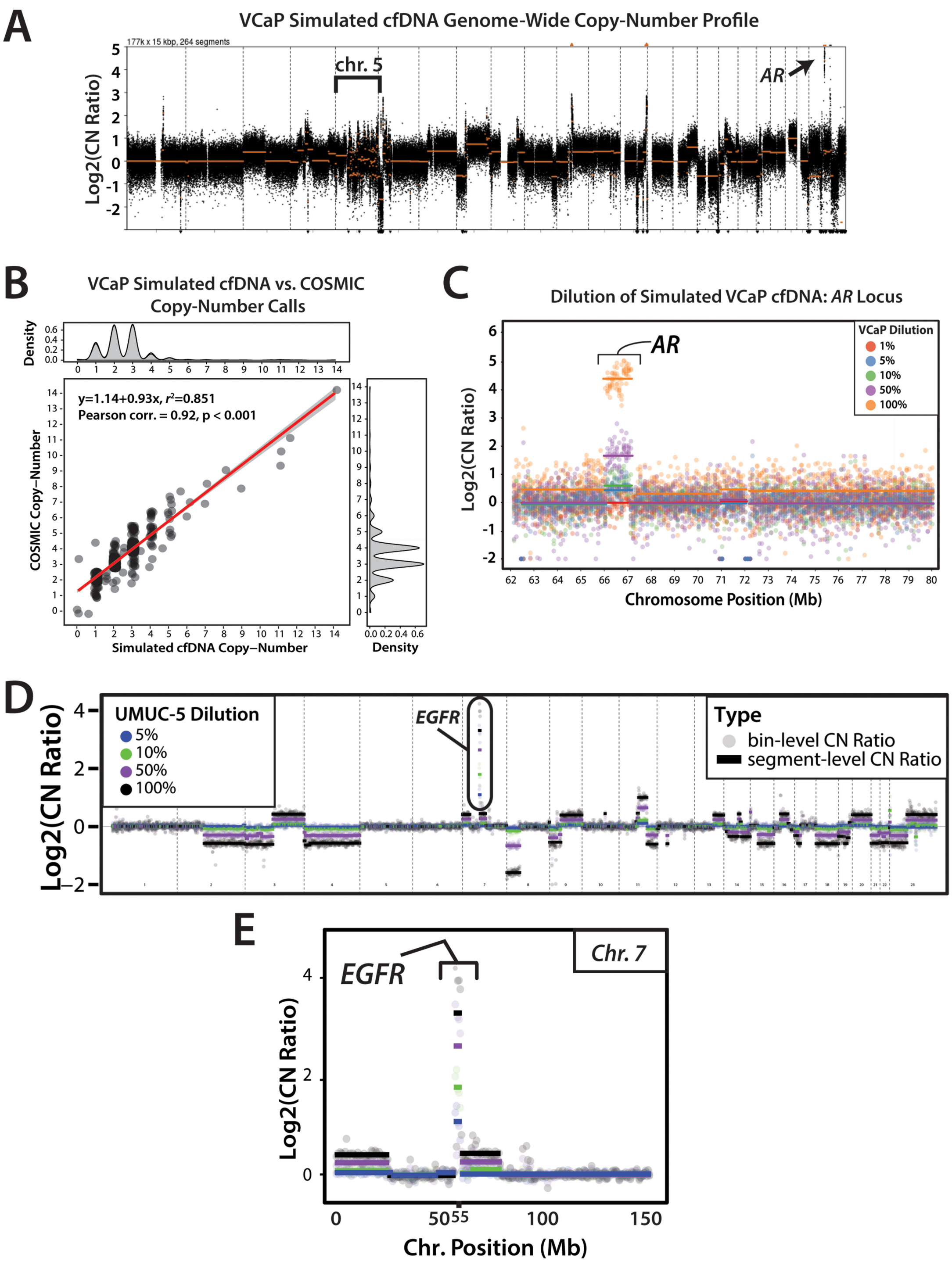

Supplementary Figure 2: Robust copy number alteration (CNA) detection by low-pass whole genome sequencing (WGS) of artificial cfDNA on bench top sequencers. (**A**) Low-pass WGS generated genome wide copy number profile of the VCaP prostate cancer cell line using sheared genomic DNA to simulate cfDNA. The known focal *AR* amplification on chr X and the chromothriptic event on chr 5 are indicated. Bin-level estimates are plotted as black dots, and segmented copy-number calls are plotted as orange lines. (**B**) Correlation of integer copy number values from low-pass WGS artificial cfDNA to reported VCaP copy number values in the COSMIC database. *AR* copy number for unamplified VCaP was capped at 14 given variability in reported copy number (see Methods). Pearson correlation and density plots are shown. (**C**) The high-level *AR* amplification in simulated VCaP cfDNA can be detected down to 5% tumor content. *In vitro* dilution of simulated VCaP cfDNA to the indicated tumor content using was performed using a healthy male control cfDNA sample prior to low-pass WGS. Log (base 2) copy number ratios (Log2 CN Ratio) are plotted. (**D**) Bin-level and segmented genome-wide copy number calls from a similar *in vitro* dilution series of simulated UMUC-5 (a urothelial cancer cell line) cfDNA subjected to low-pass WGS. Broad whole-chromosome and arm-level copy-number alterations, including both 1- and 2-copy deletions, are called at expected log2CN values across dilutions. The *EGFR* locus is highlighted. (**E**) The known focal *EGFR* amplification is clearly detected down to effective tumor content of 5%. Bin sizes: 15 Kbp (A & C) or 1Mbp (D & E). Segmentation p-value threshold: 0.01.

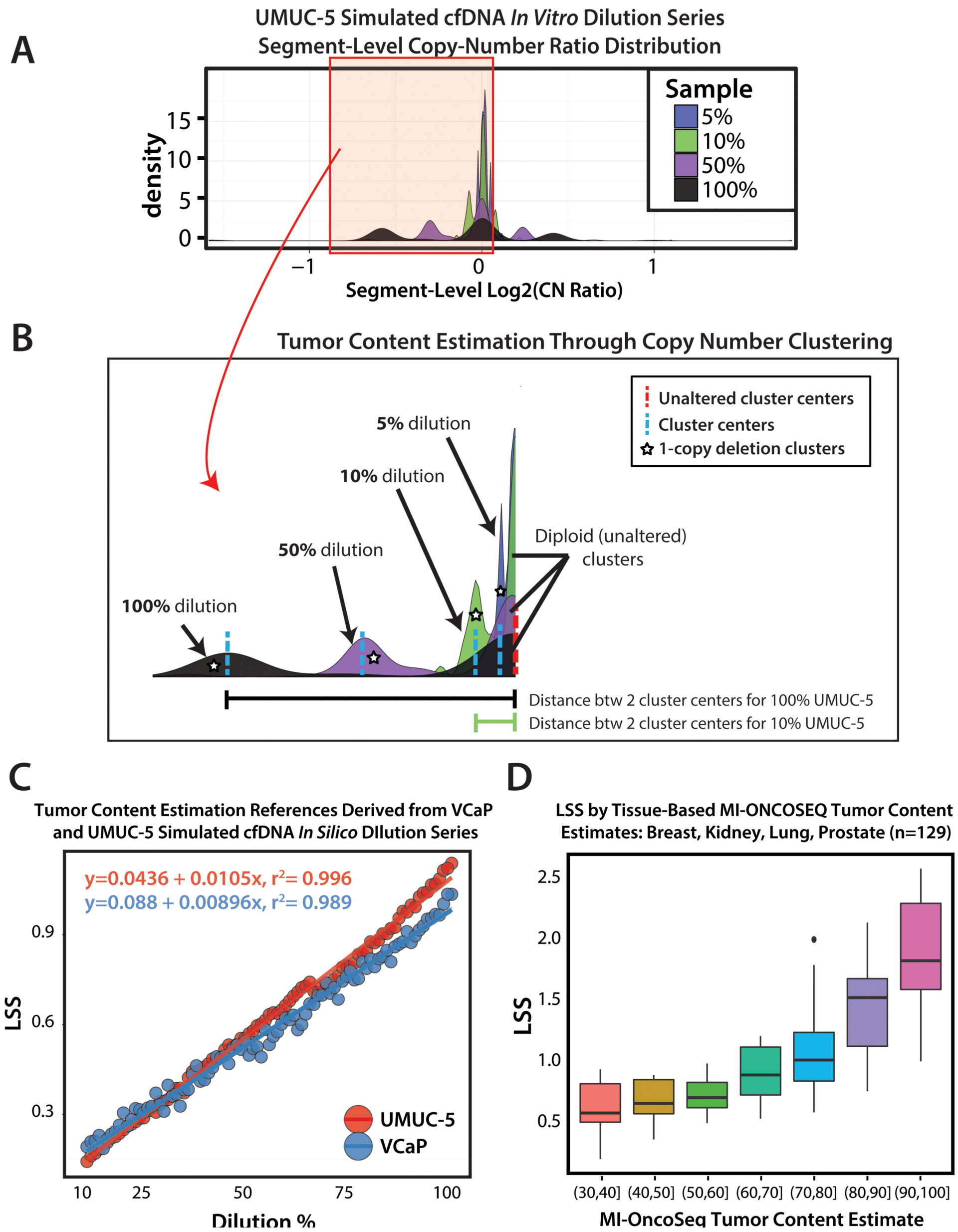

Supplementary Figure 3: Cell free DNA (cfDNA) tumor content approximation from low-pass whole genome sequencing (WGS) derived copy number profiles. Unlike in tissue based next generation sequencing (NGS), tumor content cannot be assessed a priori for cfDNA. Such information is critical to guide sequencing depth. Hence, most cfDNA approaches employ ultra-deep, high fidelity sequencing at limited loci to guide therapy with or without direct tumor content approximation. Here we leverage the near ubiquity of copy number alterations (CNAs) across tumors and our ability to rapidly generate whole genome copy number profiles from cfDNA subjected to low-pass WGS to estimate cfDNA tumor content based on the distribution of segment-level copy-number calls as part of our PRINCe workflow. (**A**) The relative density of segment-level log (base 2) copy-number ratio (log2CN) values from low-pass WGS of the *in vitro* simulated UMUC-5 cfDNA dilution series (samples according to the legend). (**B**) The basic principles of copy-number clustering and tumor content approximation as part of the PRINCe workflow are shown using the density of segment-level log2 copy-number ratio values for the simulated UMUC-5 cfDNA *in vitro* dilution series (from highlighted region of A). Clusters are called using a mean-shift clustering algorithm on segmented log2CN values, and cluster centers are used to determine a least-squares distance metric (LSS) for tumor content approximation (see Methods, Supplementary Figure 2). Cluster assignment for presumed 1-copy deletions detected by low-pass WGS in the UMUC-5 simulated cfDNA dilution series are labeled (stars), as are 1-copy deletion (blue dashed vertical line) and diploid/unaltered (red dashed vertical line) cluster centers. As tumor content decreases so does the distance between cluster centers. Aggregate distance between all cluster centers for a given cfDNA sample is calculated (as LSS) and translated to estimate the cfDNA tumor content. (**C**) Tumor content approximation from segmented log2CN calls (bin size: 1Mbp; segmentation p-value threshold: 0.01) across *in silico* dilution of simulated VCaP and UMUC-5 cfDNA were used to establish reference distributions for LSS interpretation and tumor content approximation. (**D**) Validation of our LSS based tumor content approximation approach on segmented whole exome sequencing based copy number profiles from 129 advanced/metastatic cancer (prostate, kidney, lung and breast cancer) tissue samples sequenced as part of the MI-ONCOSEQ program. Box-plots of our LSS metric stratified by MI-ONCOSEQ estimated tumor contents (through modeling SNVs and heterozygous SNPs, lower estimate is 30%) are shown.

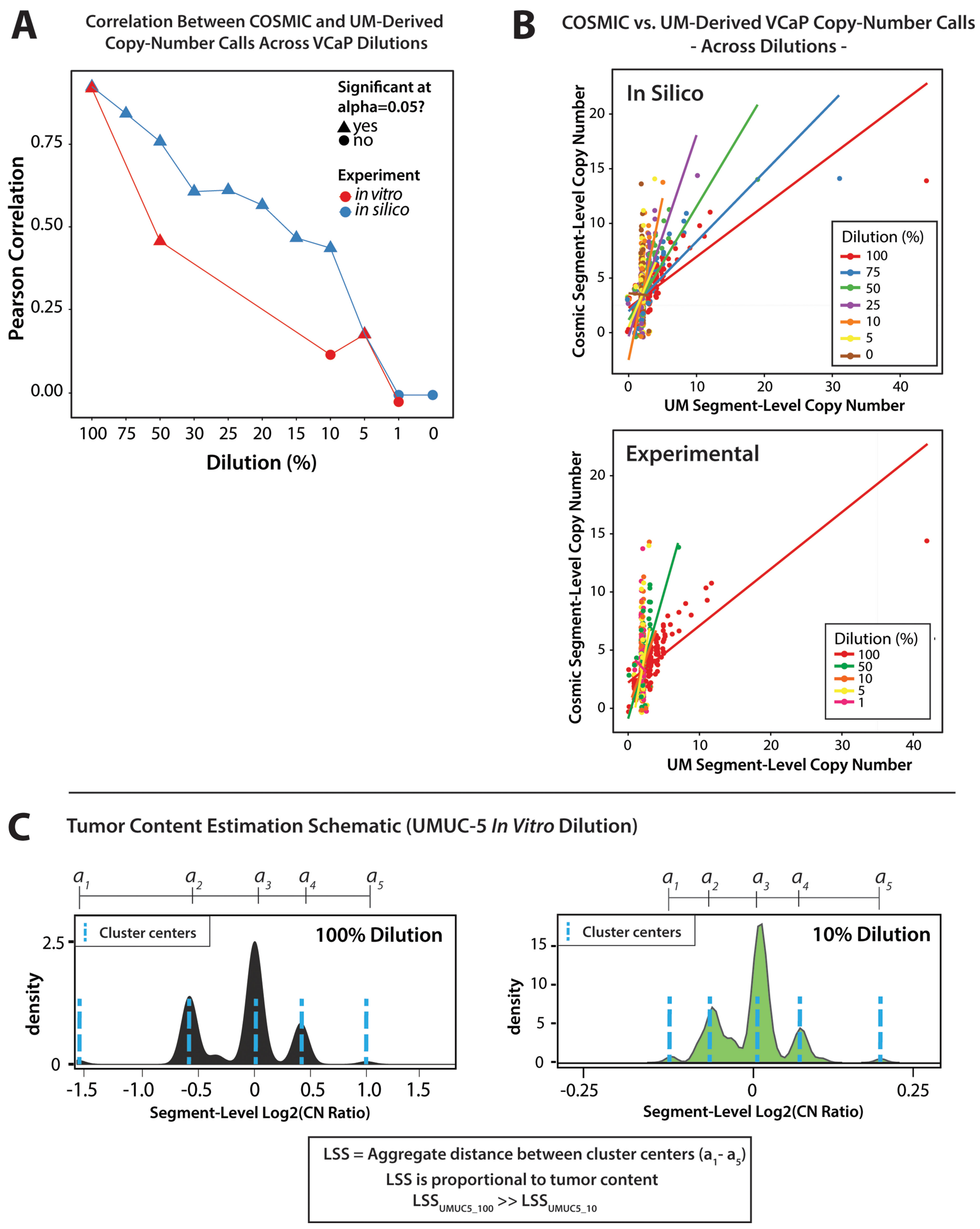

Supplementary Figure 4: Validation of low-pass WGS copy number estimation for use in cfDNA tumor content estimation. (**A**) Correlation between low-pass WGS and COSMIC copy number calls for *in vitro* and *in silico* dilutions of simulated VCaP cfDNA. Copy number analysis was performed on data from low-pass whole-genome sequencing (WGS) of *in vitro* and *in silico* dilution series for simulated VCaP cfDNA (see Methods). Pearson correlations between COSMIC integer-level segmented copy-number and low-pass WGS copy-number (UM-Derived) values are shown across select *in silico* and all *in vitro* dilutions. (**B**) Scatterplot of COSMIC integer-level copy number values compared to UM-Derived values for select *in silico* and all *in vitro* dilutions. Points are colored by dilution, and fitted linear regression lines are plotted for each dilution. (**C**) Key parameters for cfDNA tumor content estimation based on WGS copy-number profiles. Relative density of segment-level log (base 2) copy number ratio (Log2[CN Ratio]) values from low-pass WGS of UMUC-5 simulated cfDNA is plotted separately for 100% (undiluted) and 10% dilutions. Hypothetical cluster centers are denoted as blue dashed vertical lines, and correspond to elements *a1 - a5* labeled above each plot. A least-squares distance metric (LSS) is calculated (see Methods) from cluster centers assigned via a mean-shift clustering algorithm, and LSS is translated to approximate cfDNA tumor content. LSS is proportional to approximate tumor content, with larger LSS values representing higher effective cfDNA tumor content.

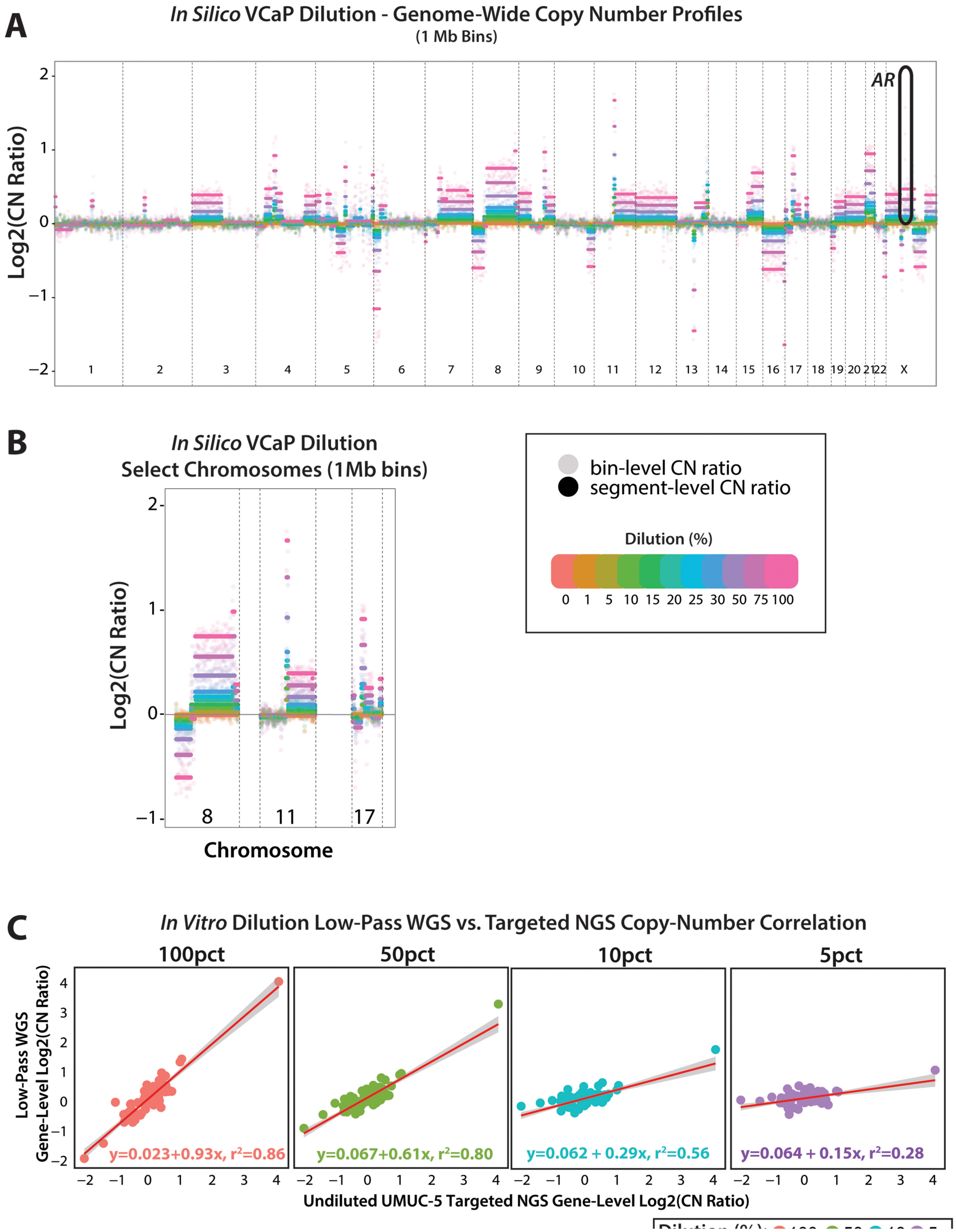

Supplementary Figure 5: Genome-wide low-pass whole genome sequencing (WGS) copy number calls for in silico dilution of simulated VCaP and UMUC-5 cfDNA. (**A**) Whole-genome bin- (gray points) and segmented (colored bars) copy-number calls (bin size: 1 Mb, segmentation p-value threshold: 0.01) at select *in silico* dilutions for VCaP low-pass WGS data highlight log (base 2) copy number ratio values (Log2 CN Ratio) values at expected gradations across dilutions for alterations > 2 Mbp in length. The known focal *AR* amplification in VCaP is ~1Mbp in length (COSMIC *AR* amplification call: chrX:66031108-67075149) and can be seen via bin-level estimates at *AR* loci as shown. (**B**) Zoomed view of bin- and segmented copy-number calls for chromosomes 8, 11, and 17 shows both broad and focal copy number alterations at Log2 CN Ratios consistent with *in silico* dilution. (**C**) Comparison of gene-level Log2CN values from targeted NGS of undiluted, unamplified simulated UMUC-5 cfDNA and low-pass WGS gene level calls for *in vitro* UMUC-5 dilution (see Methods). Points are colored by *in vitro* dilution, and fitted linear regression lines and 95% confidence intervals are plotted. Linear models and r2 values are provided for each *in vitro* dilution.

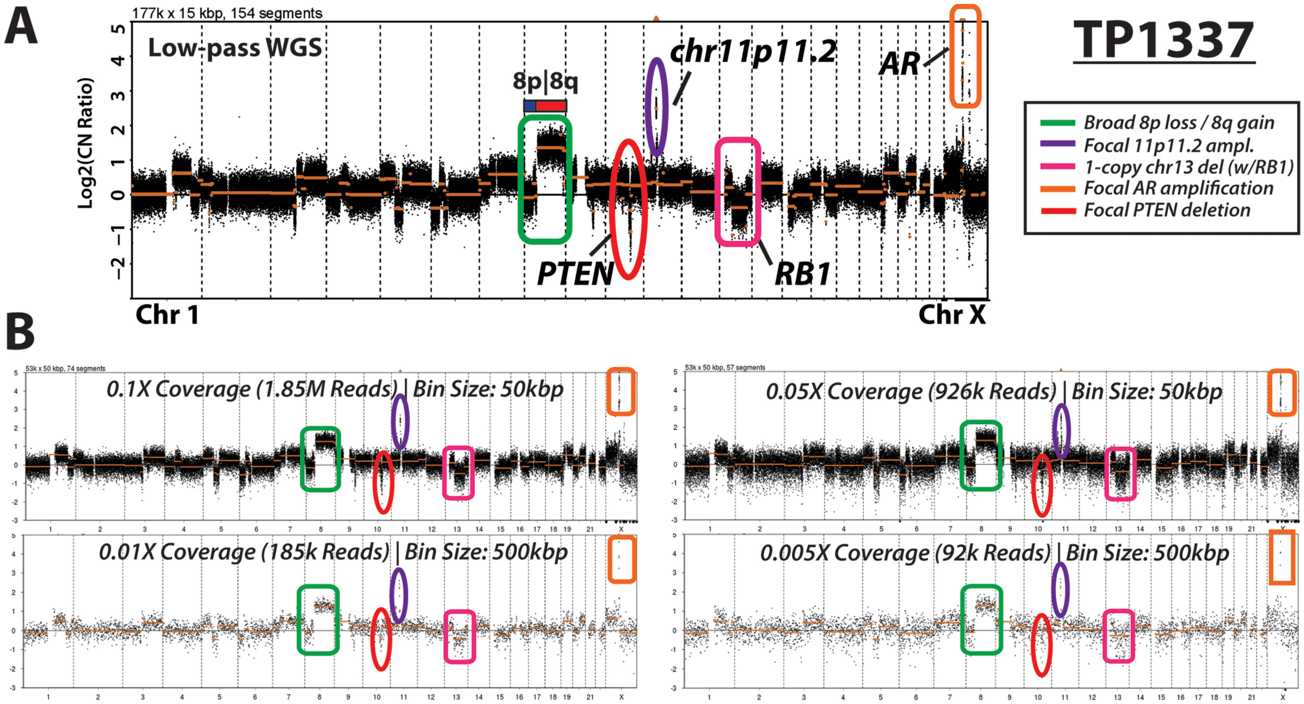

Supplementary Figure 6: Bioinformatic analysis highlighting potential feasibility of ultra-low pass (<0.01x) whole genome sequencing (WGS) of cfDNA as a disease monitoring application from cell-free DNA in patients with advanced cancer. (**A**) Genome-wide log2(CopyNumberRatio) (Log2CN) calls for TP1337, a high tumor content cfDNA sample from a patient with mCRPC, are displayed for low-pass WGS data (0.82x whole-genome coverage). Key copy-number alterations detected are circled, including broad gain of 8q (green), focal amplification of chr11p11.2 (purple) and *AR* (orange), and focal deletions of *RB1* (1-copy; pink) and *PTEN* (2-copy; red). (**B**) *In silico* downsampling experiments highlight the ability to detect both focal and broad copy-number alterations from TP1337 cfDNA WGS data at whole-genome coverages down to 0.005 x. Bin size and number of high-quality (MAPQ ≥ 37) mapped reads used for copy-number analysis are indicated at each coverage, and regions affected by copy-number alterations detected in original low-pass WGS are circled.

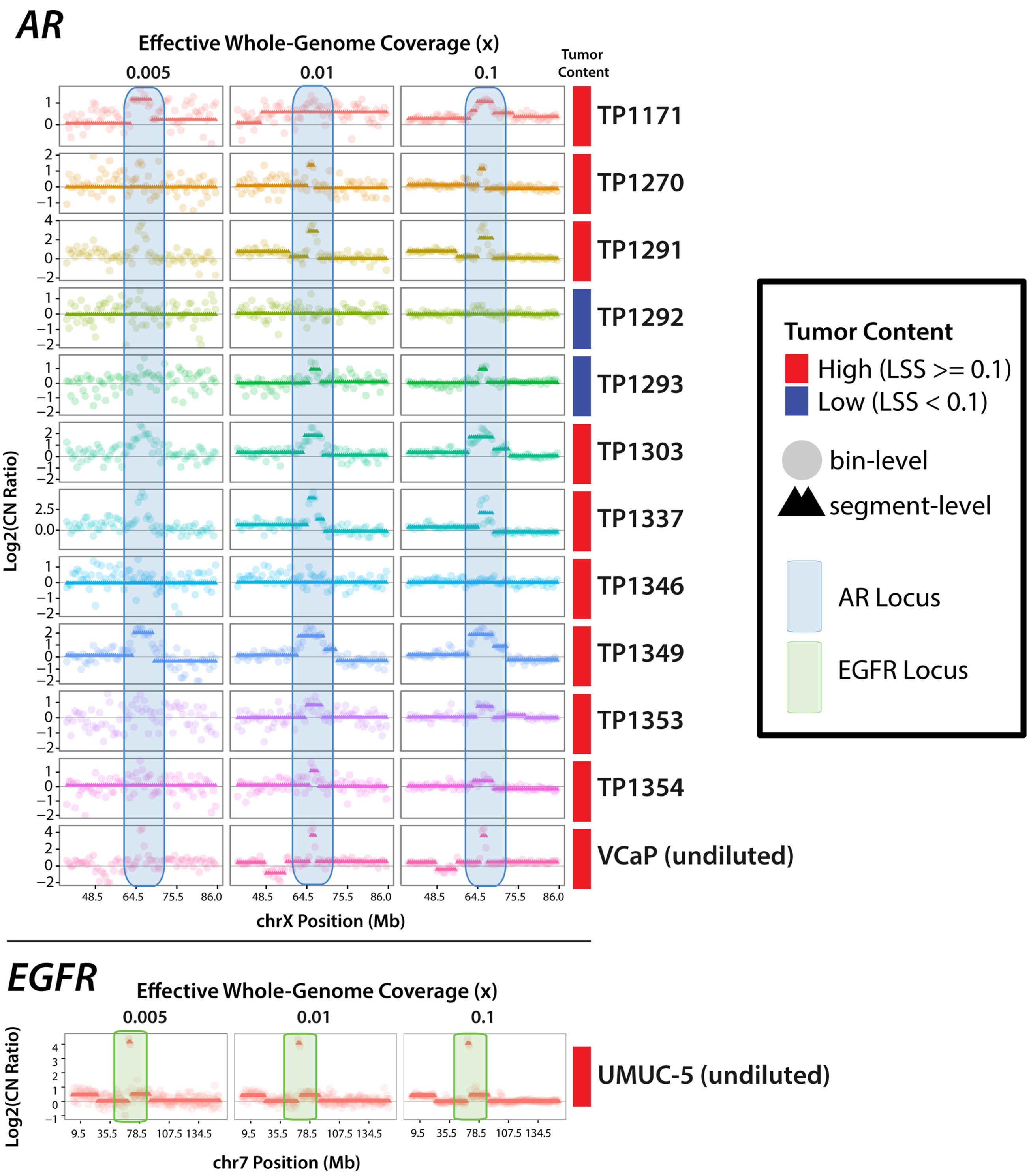

Supplementary Figure 7: AR and EGFR amplifications detected in in silico downsampling of simulated cell line cfDNA and patient cfDNA samples. *In silico* downsampling experiments were carried out on low-pass whole genome sequencing (WGS) data from simulated cell line cfDNA (VCaP and UMUC-5) and 11 cfDNA samples from patients with mCRPC to yield ultra low effective whole genome coverages (0.1x, 0.01x, and 0.005x). (**A**) Bin- and segment-level log (base 2) copy number ratio (Log2 [CN Ratio]) calls are presented across effective whole genome coverages in *AR* region on chrX for mCRPC samples with detectable *AR* amplifications by low-pass WGS as well as the undiluted simulated VCaP cfDNA sample. Points are colored by sample, 500kbp bin-level and segment-level Log2 (CN Ratio) estimates are represented by lightly shaded circles and densely colored triangles, respectively. Tumor content estimates are highlighted by red (high) and blue (low) boxes at right. The *AR* locus is highlighted in light blue boxes. (**B**) Bin- and segment-level Log2 (CN Ratio) copy number calls for undiluted artificial UMUC-5 cfDNA sample are presented across effective whole-genome coverages for chr7, and the *EGFR* locus is highlighted in light green boxes. Bin- and segment level estimates are indicated as in A.

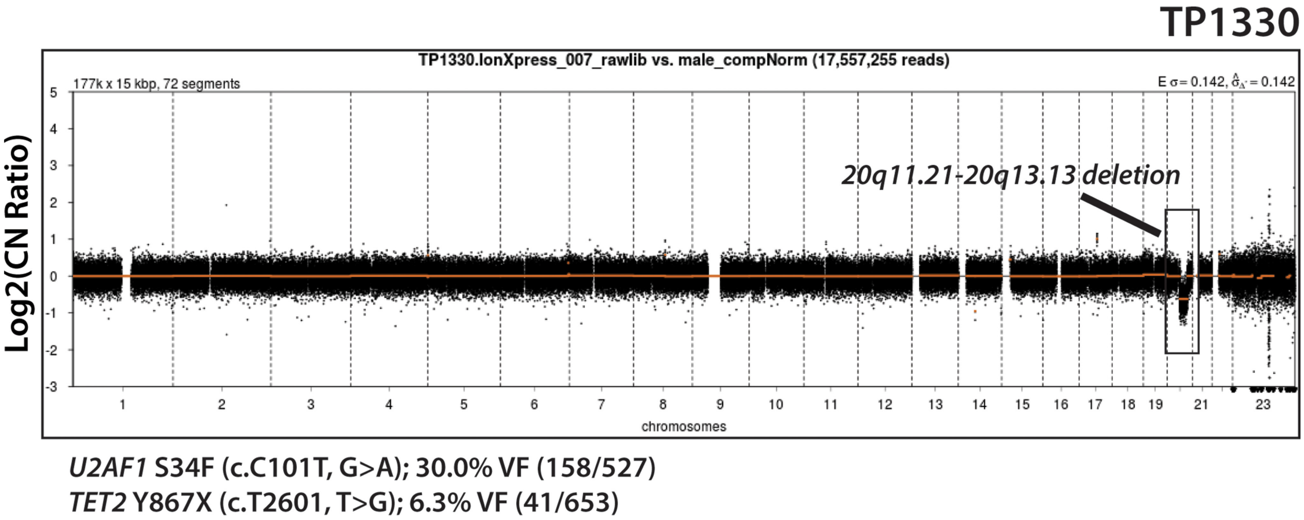

Supplementary Figure 8: PRINCe assessment of sample from patient with metastatic castration-resistant prostate cancer (mCRPC) identifies unique molecular alterations consistent with contaminating cell-free DNA from white blood cells in the context of concomitant myelodysplastic syndrome. Low-pass whole-genome sequencing (WGS) copy-number calls (bin size: 15kbp, segmentation p-value threshold: 0.01) are plotted for a cfDNA sample from TP1330, a patient with mCRPC. A unique 19 Mbp deletion (affecting chr20q11.21-20q13.13) is present on chr20, with no other copy-number alterations detected genome-wide. By targeted NGS of unamplified residual cfDNA for this same sample, we identified a *U2AF1* S34F hotspot mutation (30% variant fraction (VF), 527 covering reads) that in combination with the chr20 deletion is strongly suggestive of contaminating cell-free DNA (likely from white blood cells) in the context of concomitant myelodysplastic syndrome, consistent with clinical reports of anemia, and potentially arising in response to prior therapy.

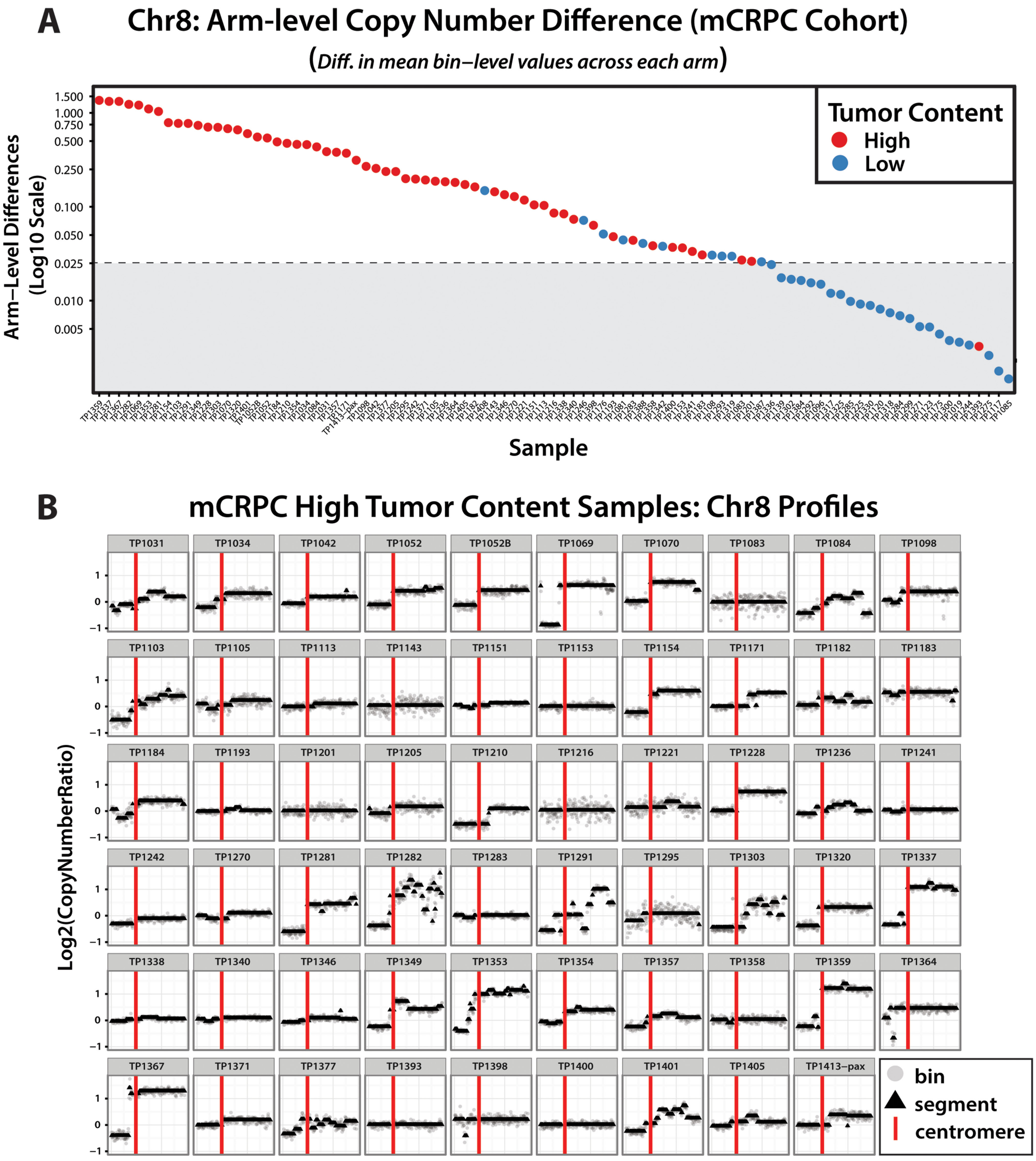

Supplementary Figure 9: Low-pass whole genome sequencing (WGS) copy number profiles from cell-free DNA (cfDNA) in patients with metastatic castration-resistant prostate cancer (mCRPC) highlight detection of arm- and sub-arm level copy-number alterations on chromosome 8 (chr8), even at low cfDNA tumor contents. (**A**) Points representing chr8 'difference values' (the absolute value of the difference in mean bin-level log2 copy-number estimates between p and q arm of chr8) for all samples in the mCRPC cohort (n = 93). Samples are sorted in order of descending difference value, and colored by cfDNA tumor content as assigned by LSS analysis (red = High (LSS ≥ 0.1; blue=Low (LSS < 0.1)). A threshold of 0.025 was applied to difference values to determine whether each cfDNA sample had detectable chr8 copy number alterations (≥ 0.025 = 8p or 8q copy-number alterations) consistent with copy number events known to occur early in prostate cancer progression. (**B**) Low-pass WGS chr8 copy-number profiles for all high tumor content cfDNA samples (n = 59) from men with mCRPC. As indicated, gray dots correspond to bin-level copy-number estimates, while black triangles denote the segment-level copy number value for the corresponding bin. The vertical red line in each plot indicates the centromere region to aid in p- and q-arm determination.

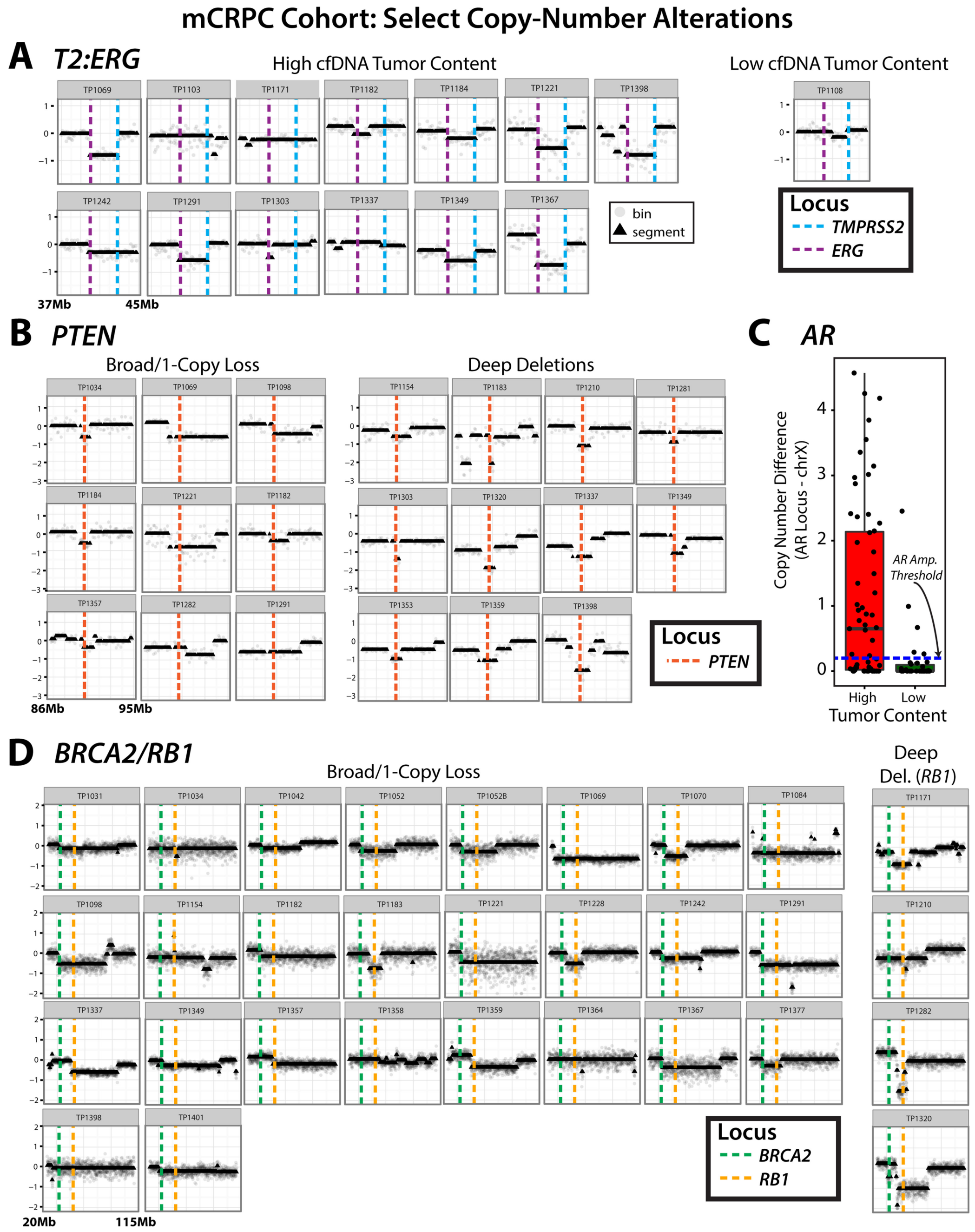

Supplementary Figure 10: Clinically relevant somatic copy number alterations detected via low-pass whole genome sequencing (WGS) of cell-free DNA (cfDNA) in patients with metastatic castration-resistant prostate cancer (mCRPC). (**A**) Bin- and segment-level copy-number values from low-pass WGS data in 14 cfDNA samples from patients with mCRPC that have copy-number alterations or breakpoints on chr21 consistent with genomic events leading to TMPRSS2:ERG or ETS family gene fusions (displayed region: chr21:37.0–45.0 Mb). Tumor content for the corresponding cfDNA sample is listed at top. Dashed vertical lines at 40Mb (purple) and 42.8 Mb (cyan) represent loci corresponding to *ERG* and *TMPRSS2* coding sequence (hg19 reference coordinates), respectively. (**B**) Bin- and segment-level copy-number values from low-pass WGS data in 20 high tumor content cfDNA samples from patients with mCRPC with chr10 copy-number deletions affecting the *PTEN* locus (displayed region: chr10:86.0-95.0Mb). Samples are grouped by deletion type (broad/1-copy or deep). A dashed vertical line at 90 Mb (orange) represents the location of *PTEN* coding sequence (hg19 reference coordinates). (**C**) Combined box and scatterplot for *AR* deviance values (mean 100kb bin-level log2 copy-number estimates at *AR* locus [chrX:66.0-67.5Mb] minus median 100kb log2 bin-level copy number estimates across chrX q-arm) used to identify focal *AR* amplifications in our mCRPC cohort (see Supplementary Methods). Samples with deviance values ≥ 0.2 were considered positive for *AR* amplification, and this threshold is represented by the blue horizontal dashed line as annotated on the plot. Combined box and scatter plots are plotted separately for high (red) and low (green) cfDNA tumor content. (**D**) Bin- and segment-level copy-number values from low-pass WGS data in 30 high tumor content cfDNA samples from patients with mCRPC with chr13 copy-number deletions affecting *BRCA2/RB1* loci (displayed region: chr13:20.0-115.0 Mb). Samples are grouped by deletion type (broad/1-copy vs deep deletion), and dashed vertical lines at 33Mb (green) and 49 Mb (yellow) indicate *BRCA2* and *RB1* loci, respectively. Reference genome coordinates: hg19. Bin-width: 100 kb. Copy number segmentation switch-point threshold (*p*-value): 0.01.

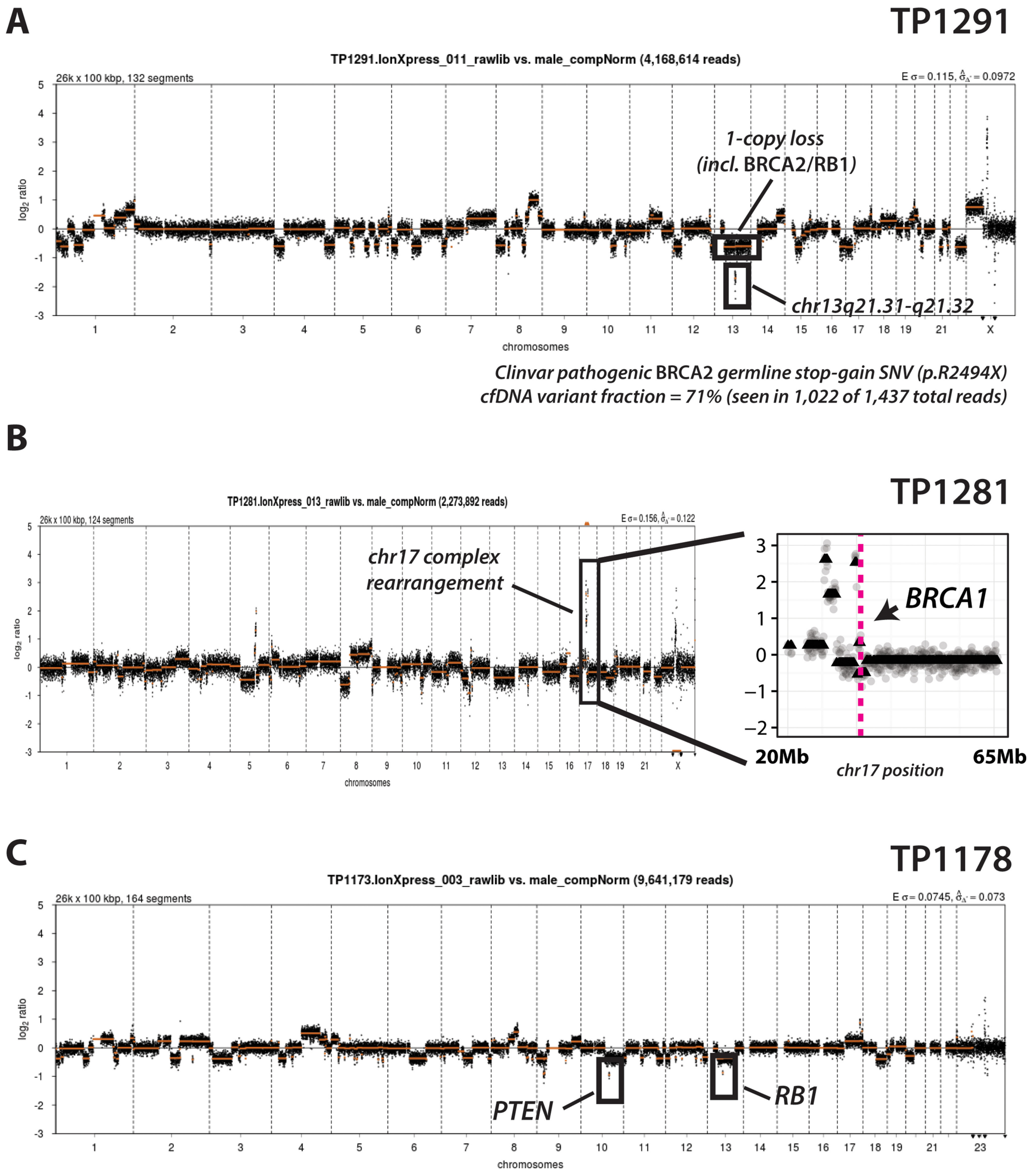

Supplementary Figure 11: Low-pass whole genome sequencing (WGS) of cell-free DNA (cfDNA) identifies likely copy-number alteration affecting BRCA1 and BRCA2 in patients with mCRPC as well as clinically relevant alterations (including focal PTEN and RB1 deletions) in treatment-naïve patient with aggressive disease. (**A**) Genome-wide bin- (black dots) and segment-level (orange lines) log2 copy number estimates from low-pass WGS sequencing data for TP1291, a patient with mCRPC who progressed rapidly through treatment with abiraterone, enzalutamide, docetaxel, and cabazitaxel over the course of 11 months preceding cfDNA sample collection. Broad 1-copy loss on chr13 (including *BRCA2* and *RB1*) is indicated, as is the focal 2-copy deletion of a nearby loci absent any coding transcripts (chr13q21.31-q21.32). Targeted NGS of paired unamplified cfDNA for TP1291 identified a germline Clinvar pathogentic *BRCA2* stop-gain SNV (p.R2494X; variant fraction = 71%,with 1,437 total covering reads), suggesting biallelic inactivation of *BRCA2* through copy-number deletion of the non-mutated copy of *BRCA2*. (**B**) Bin- (black dots) and segment-level (orange lines) log2 copy number estimates from low-pass WGS sequencing data are presented genome-wide at for a 45Mb section of chr17 for TP1281, a sample from a patient with mCRPC. Region affected by putative complex rearrangement on chr17 is highlighted on the genome-wide plot, and at right, a zoomed version indicates the location of *BRCA1* (dashed vertical pink line; hg19 reference coordinates). (**C**) Genome-wide bin- (black dots) and segment-level (orange lines) log2 copy number estimates from low-pass WGS sequencing data (0.52×, 14.2 million reads) for TP1178, a cfDNA sample from a treatment-naïve patient with mCRPC. Focal deep deletions of *PTEN* and *RB1* are identified in addition to multiple arm- and sub-arm level copy number gains or losses genome-wide, suggesting potential clinical utility for PRINCe assessment in treatment-naïve patients with advanced cancer and/or likely to have high disease burden. Bin width: 100 kbp.

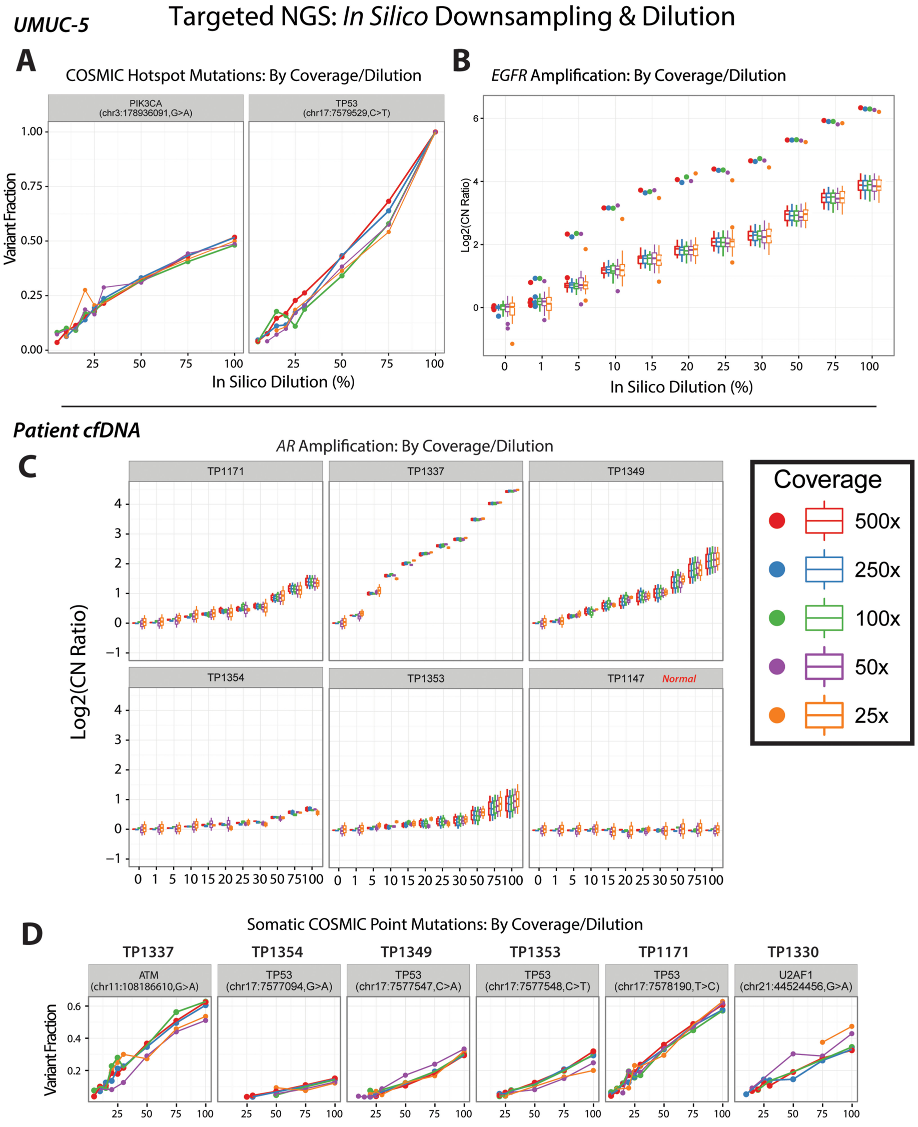

Supplementary Figure 12: Automated point mutation and copy number alteration calls across in silico dilution and downsampling of targeted next generation sequencing (NGS) from simulated cell line cfDNA and patient cfDNA samples. *In silico* dilution and downsampling experiments were carried out on targeted NGS data for unamplified, undiluted genomic DNA (gDNA) from the UMUC-5 cell line, as well as unamplified aliquots of 10 patient cfDNA samples (5 high tumor content mCRPC samples, 1 mCRPC sample with germline chr20 deletion, and 4 male normal controls). These samples were sequenced using the DNA component of the Oncomine Comprehensive Assay (OCP), a targeted NGS panel comprised of 2,530 amplicons targeting 126 genes, including oncogenes, tumor suppressors, and copy-number targets recurrently altered across cancers. *In silico* dilutions were carried out at all integer-level dilutions (0-100%) across 5 different effective coverage thresholds (500×, 250×, 100×, 50×, and 25×) (see Methods). (**A**) Analyses of select *in silico* dilution and downsampling data from two COSMIC hotspot point mutations detected in targeted NGS of undiluted simulated UMUC-5 cfDNA are presented. A heterozygous nonsynonymous *PIK3CA* hotspot mutation (p.E545K, detected at 49.6% (FAO/FDP: 916/1848)) and homozygous stop-gain *TP53* hotpot mutation (p.W53X, 100% (668/668)) are reliably detected at expected variant fractions across targeted NGS coverages as low as 50x down to effective tumor contents of 10-15%. (**B**) Box-and-whisker plots of amplicon level log base 2 copy number ratio (Log2 [CN Ratio]) estimates from OCP sequencing of undiluted simulated UMUC-5 cfDNA are plotted for all OCP *EGFR* target amplicons (n=33) across select *in silico* dilutions and coverages. Known focal *EGFR* amplification (undiluted UMUC-5 OCP gene-level *EGFR* Log2 (CN Ratio) value = 3.89) in UMUC-5 cell line is reliably detected (median Log2 [CN Ratio] ≥ 0.6; see Methods) across coverages down to 25x at 5% effective tumor content. (**C**) Box-and-whisker plots of Log2 (CN Ratio) values for all OCP *AR* amplicons ($n = 17$) are shown across in silico dilutions and coverages for 6 patient cfDNA samples (5 mCRPC samples with detectable AR amplifications by low-pass whole genome sequencing (WGS) copy-number analysis, and 1 control sample). Depending on starting tumor content of undiluted ('100%' dilution) patient cfDNA samples, *AR* amplifications can be reliably detected in targeted NGS data from CPRC cfDNA samples at coverages down to 25x at 5% dilution. (**D**) Putative clonal somatic COSMIC hotspot mutations detected via targeted NGS in 6 mCRPC patient samples are plotted across select *in silico* dilutions and effective coverages. All mutations are detected at heterozygous variant fractions in undiluted ('100%' dilution) OCP targeted NGS data, adjusting for cfDNA tumor content. Depending on starting (undiluted) cfDNA tumor content, hotspot mutations were reliably detected by OCP targeted NGS at coverages as low as 50x with 15% effective tumor content.

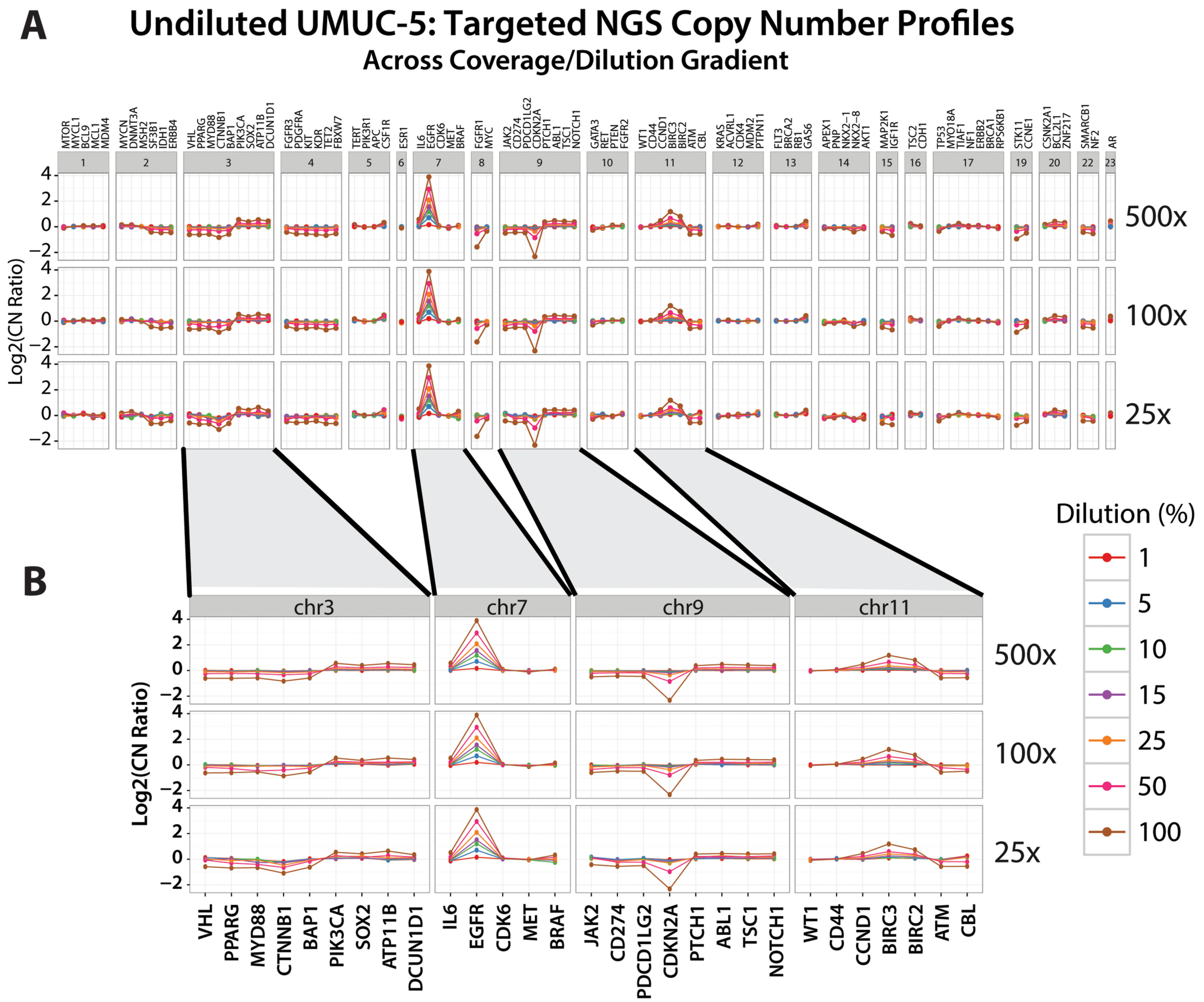

Supplementary Figure 13: Targeted NGS gene-level copy-number analysis across in silico dilution and downsampled coverages for simulated UMUC-5 cfDNA. (**A**) Gene-level log (base 2) copy number ratio (Log2 CN Ratio) values derived from Oncomine Comprehensive Assay (OCP) targeted NGS data for simulated UMUC-5 cfDNA are plotted across select *in silico* dilutions at three separate coverages (500×, 100×, and 25×) for all OCP target genes with at least 3 target amplicons (n = 90). Points represent gene-level Log2CN Ratio values, with points (and lines connecting points) colored by *in silico* dilution proportion. The known focal *EGFR* amplification can be seen as peak on chromosome 7. (**B**) Zoomed view of OCP gene-level Log2CN Ratio values for select chromosomes (chr3, chr7, chr9, and chr11). Focal amplifications or deletions identified by low-pass WGS can be detected at targeted NGS coverages down to 25× fordilutions with as low as 5% effective tumor content.

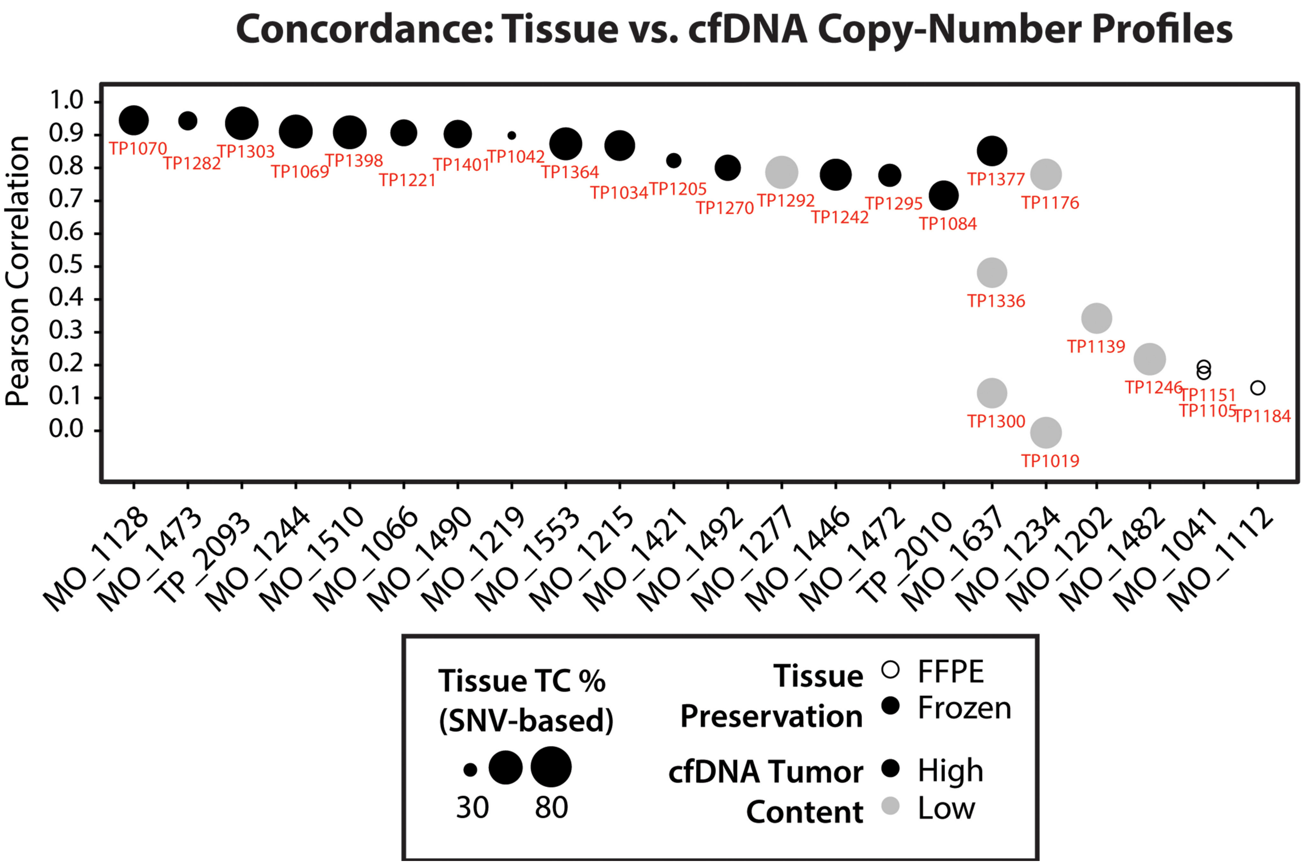

Supplementary Figure 14: Genome-wide copy number profile concordance for cfDNA low-pass whole genome sequencing (WGS) as compared to patient-matched tissue whole exome sequencing (WES) copy-number profiles. Pearson correlation coefficients are plotted for genome-wide segmented copy-number profiles from 23 patients with comprehensive tissue NGS profiling and PRINCe assessment of at least 1 cfDNA sample (see Supplementary Methods). As indicated, each point represents the correlation of a single cfDNA sample's low-pass WGS genome-wide profile as compared to the patient-matched whole exome sequencing tissue copy-number profile (see Supplementary Methods). The size of each point corresponds to the SNV-based estimated tissue tumor content (which varies from 30 to 80%), while the color represents cfDNA tumor content (black: high; gray: low). Circle filling represents patient-matched tissue preservation type (filled: frozen; unfilled: FFPE). cfDNA sample identifers are provided in red.

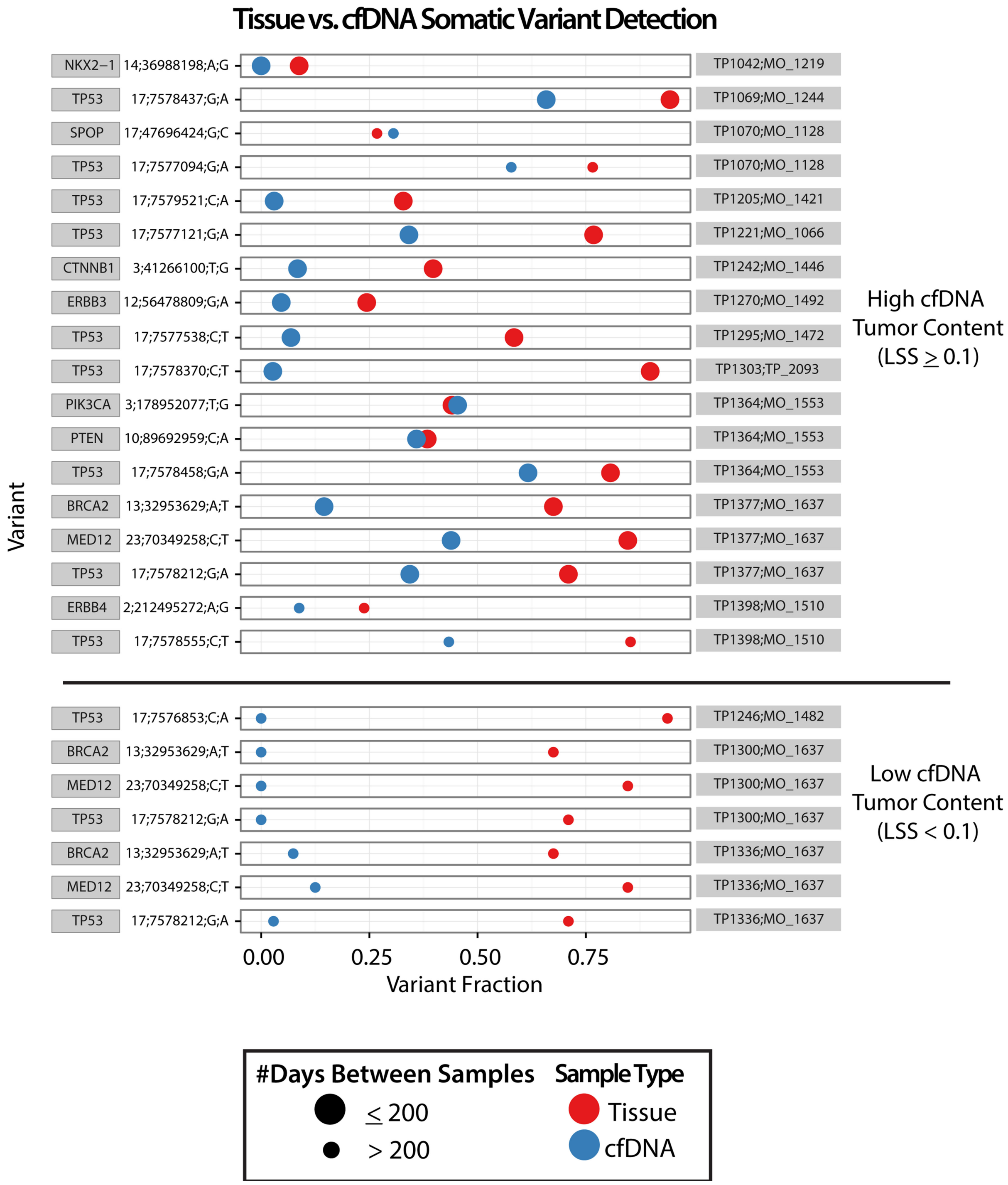

Supplementary Figure 15: Somatic point mutation concordance between tissue and cell-free DNA (cfDNA) mutation analyses. Tissue and cfDNA variant fractions are plotted for 26 point mutations identified in patient-matched tissue-based whole exome sequencing (WES) that fall in regions targeted by the Oncomine Comprehensive Assay (OCP). Variants are sorted vertically by increasing cfDNA sample identifier, and all cfDNA/tissue id combinations are listed on right hand side. Each row corresponds to a single variant detected in the comprehensive patient-matched tissue profile, and the gene, genomic coordinates, and allelic changes are indicated on left hand side of each row. For each variant, both tissue- (red) and cfDNA-based (blue) variant fractions are plotted (variant fraction of 0% = not detected), and points are sized by whether the corresponding cfDNA sample was taken within 200 days of the patient-matched tissue biopsy as indicated in the legend. Variants are grouped vertically by cfDNA tumor content for the corresponding cfDNA sample (top: high tumor content (LSS ≥ 0.1); bottom: low tumor content (LSS < 0.1)). Overall, 17 of 18 (94.4%) point mutations detected in patient-matched tissue specimens with ≥ 1 high tumor content cfDNA sample were also detected by OCP targeted NGS of the corresponding cfDNA sample.

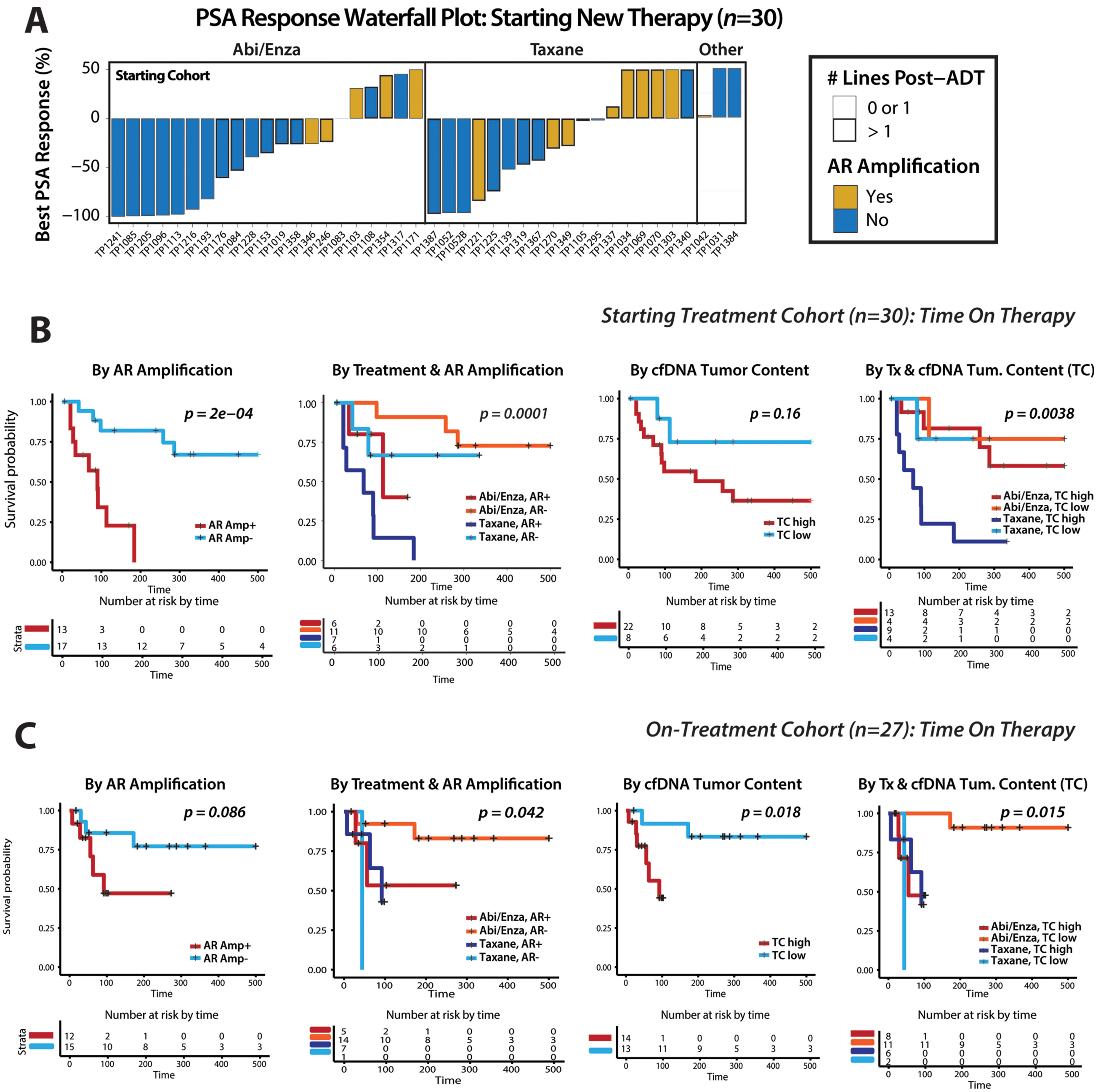

Supplementary Figure 16: PSA waterfall and outcome analyses in samples from patients starting and on therapy. Exploratory analyses of association between circulating biomarkers and outcome in patients with metastatic castration-resistant prostate cancer (mCRPC) supports cfDNA detectable AR amplification as a poor overall prognostic factor independent of treatment type. (**A**) Waterfall plot summarizing prostate specific antibody (PSA) response for all samples from men with mCRPC with complete PSA data taken between therapies (n = 42). Height of bars represent the percentage change in PSA response as calculated by subtracting the PSA level at sample date from the best PSA observed after sample date while on the current or initiated treatment, and dividing by starting PSA value. Bars are ordered horizontally within treatment category (Abi/Enza, Taxane, or Other) by PSA response. Bars are colored by cfDNA detectable AR amplification status (yellow = cfDNA detectable AR amplification; gray = no cfDNA detectable AR amplification) and bars corresponding to samples taken from men who have received more than one line of therapy post-ADT are outlined in bold. B-C. Kaplan-Meier survival curves are plotted for analyses exploring association between cfDNA detectable AR amplification or cfDNA tumor content and total time on therapy in samples taken from men with CRPC (**B**) starting treatment and (**C**) on therapy. For each subset, Kaplan-Meier time on therapy analyses are plotted separately (from left to right) for cfDNA AR amplification, treatment by cfDNA AR amplification, cfDNA tumor content, and treatment by cfDNA tumor content. Survival curves are colored by corresponding strata, and risk tables at selected timepoints are displayed below each Kaplan-Meier plot.

Supplementary Table 1: Sample index and sequencing statistics. See Supplementary_Table_1

Supplementary Table 2: Sample index and sequencing statistics. See Supplementary_Table_2

Supplementary Table 3: mCRPC patient molecular info. See Supplementary_Table_3

Supplementary Table 4: Prioritized point mutation and insertion/deletion variant calls from targeted next-generation sequencing of cell-free DNA samples. See Supplementary_Table_4

Supplementary Table 5: cfDNA vs. Tissue point mutation and indel concordance. See Supplementary_Table_5
